# Phytochemical characterization of raw and cooked traditionally consumed alimurgic plants

**DOI:** 10.1371/journal.pone.0256703

**Published:** 2021-08-26

**Authors:** Stefania Monari, Maura Ferri, Beatrice Montecchi, Mirko Salinitro, Annalisa Tassoni

**Affiliations:** 1 Department of Biological, Geological and Environmental Sciences, University of Bologna, Bologna, Italy; 2 Department of Civil, Chemical, Environmental and Materials Engineering, University of Bologna, Bologna, Italy; Bangabandhu Sheikh Mujibur Rahman Agricultural University, BANGLADESH

## Abstract

In the past, wild edible alimurgic plants became an important alternative food source when poverty, wars or drought made it difficult to access crops. These plants were considered rich in highly nutritional compounds and also frequently used as food-medicine given their health-promoting properties. With the aim of improving our knowledge on the content of beneficial or detrimental compounds in relation with past local dietary and curative traditions, 12 wild food plant species were collected from two study areas selected for their very different degree of industrialization, urbanization, and conservation of local past traditions among the population: the Bologna province (Northern Italy) and the Middle Agri Valley (Southern Italy). Protein, polyphenol flavonoid and biogenic amine (both free and conjugated) contents and antioxidant activity of raw and boiled wild food plant extracts, and of cooking water were analyzed by means of spectrophotometric and high-performance liquid chromatography methods. The results demonstrated that most of the phenolic compounds were released in the cooking water which also showed the highest antioxidant activity. Seventeen different phenolic compounds were identified, of which the health-related luteolin, luteolin-7-glucoside and rutin were the most abundant (e.g., *S*. *pratensis* L. and *C*. *intybus* L.). On the other hand, biogenic amines were absent or present at very low levels in cooking water of those very same species (e.g., *S*. *pratensis* L., *T*. *officinalis* Weber, *C*. *vesicaria* subsp. *taraxacifolia* and *C*. *intybus* L.) of which traditionally a decoction is used for therapeutic purposes. Free and conjugated spermidine and spermine were generally the most abundant biogenic amines, while none of the known detrimental monoamines (e.g., histamine) was detected. In conclusion, the present results seem to support past local popular traditions which indicated beneficial medical properties of some wild edible plant, as well as of their cooking water.

## Introduction

Before the so-called *economic boom* (1950–1970), economy and society of most European countries were mainly based on agriculture. Poverty and wars made it difficult to access crops and therefore, wild edible plants (also called *alimurgic* plants) represented an alternative food source [1]. Wild food plant foraging and consumption practices became gradually part of the traditional local knowledge, and were slowly integrated into territory customs. During the 20th century, society and people’s way of living were strongly affected by the increase in urbanization, industrialization and large-scale farming. One of the consequences was that wild food plant practices and related knowledge have been progressively disappearing. Today, however, these plant species are being revalued, receiving considerable attention from researchers and food scientists as well as from nutritionists and master chefs [1, 2].

The use of wild food plants in the Mediterranean area has been investigated previously [1, 3–5] at several sites in Albania, Greece, Italy, Spain and Morocco, pointing out that alimurgic plant consumption is strongly related to local traditions and cultural heritage. These studies also evidenced the progressive decrease of knowledge and use of these species over generations, in particular close to urban areas. Other studies were carried out in the province of Bologna (Emilia-Romagna region,) [6], one of the most economically developed regions of Northern Italy, and in the Middle Agri Valley (province of Potenza, Basilicata region, Southern Italy) [7], an area characterized by the presence of few villages with a low population density of in prevalence elderly people, and primarily sustained by agriculture. The two previous studies led to the identification of 66 and 52 wild food plants, respectively, including greens (leafy plants eaten as vegetables), fruits and semi-wild plants still known, collected and eaten (raw and/or cooked), and sometimes used for both food and therapeutic purposes (e.g., cooking water). Over time, it has been demonstrated that many edible wild plants are rich in bioactive compounds, giving them nutritional or therapeutic value. Alimurgic plants are generally characterized by low energy and high nutritional values [8]. In comparison to the corresponding cultivated species, wild food plants have a higher fiber content [9], are richer in antioxidants and flavonoids (e.g. catechins and hydroxycinnamic acids) [1, 10–13] and contain very small amounts of lipids [8]. Many of these plants were proven to have important beneficial effects, preventing chronic diseases typical of modern society, such as heart and age-related diseases, diabetes and some types of cancer [8, 14]. It should be pointed out, however, that all plants, including alimurgic ones, not only contain health-beneficial molecules but possibly also detrimental compounds, such as erucic or oxalic acids [2, 15].

Although bioactive nutraceutical and anti-nutraceutical compounds present in wild plant foods may have a great influence on human health, their metabolomic profile and complexity has yet to be fully described, even though several studies focusing on particular classes of compounds or nutritional factors have been performed [1, 16]. Among the anti-nutraceutical compounds, amines are basic nitrogenous compounds synthesized by similar metabolic pathways in plant, animals and microbial cells, and present both in free and bound forms [17, 18]. The term *biogenic amines* refers to monoamines, such as histamine (HIM), serotonin (SER), tyramine (TYM), tryptamine (TRYPT), but also aliphatic polyamines, such as diamine-propane (DAP), putrescine (PUT), cadaverine (CAD), spermidine (SPD) and spermine (SPM). In food and beverages, biogenic amines are formed after decarboxylation of precursor amino acids, either by enzymes present in the raw material and/or by microbial enzymes, and their concentration is particularly high in all those foods that are produced by fermentation processes such as cheese, wine, beer, sauerkrauts [19, 20]. Some types of biogenic amines (such as HIM, TYM, TRYPT and CAD) may cause headaches, respiratory distress, heart palpitation, hypertension or hypotension, and several allergenic disorders [20] if absorbed at too high concentrations and, therefore, they are undesirable in all foods. Aliphatic polyamines (such as PUT, SPD and SPM) are considered to be bioregulators of numerous cell functions, such as cell growth, division and differentiation processes, tissue repair and intracellular signaling, although, at high concentrations, they may sustain cancer cell proliferation. Furthermore, it has been reported that a low polyamine diet can reduce cancer growth [20] and, therefore, assessing the everyday dietary intake of biogenic amines could represent an important way of reducing their level in the body pool. To date, no data related to bound biogenic amine content of alimurgic plants, have been reported.

Interestingly, phenols have been found to interact with biogenic amines and their metabolic pathways. Catechins were observed to affect some biogenic amine biosynthetic enzymes [21]. For example, epigallocatechin gallate (EGCG) is able, at the same time, to inhibit histidine decarboxylase (HDC) and ornithine decarboxylase (ODC) activities (leading to a decrease in HIM and PUT biosynthesis) and to enhance SPD/SPM N^1^-acetyltransferase (SSAT) activity (promoting polyamine catabolism) [17, 21, 22].

Processing practices may modify the biochemical profile of foods. The effect of cooking on the biological, chemical and physical properties of vegetables has been reported for several cultivated plants [23], although few data are available for the alimurgic plant species analyzed in the present study [24]. These plants are consumed raw and/or after being cooked in different ways according to local culinary tradition or personal taste. In general, food processing not only improves flavor, digestibility and palatability, but also increases food safety through inactivation of anti-nutritional factors and destruction of microorganisms. From a nutritional point of view, the cooking process can cause thermal degradation of phytochemicals, but it can also enhance their concentration and bioavailability and the release of fiber-bound forms. Both positive and negative effects have been reported in the literature, in particular regarding polyphenol content and antioxidant capacity, while very few data presently exist regarding biogenic amines [23, 25].

The purpose of the present research was to improve the knowledge regarding the content of several phytochemicals (beneficial or detrimental) in selected wild edible plants traditionally consumed in two different Italian areas [6, 7]. Based on previous studies, twelve alimurgic species were collected and a boiling treatment was adopted to simulate one of the most common way of dietary consumption by traditional users of wild food plants. Biochemical profiles were obtained from raw and cooked plants, and from cooking water. Changes in the phytochemical composition in relation to preparation procedures are discussed.

## Materials and methods

### Materials

Twelve wild food plants were selected among the species described in two previous ethnobotanical survey studies [6, 7]: six from the area of Bologna (Emilia Romagna region, Italy), namely *Salvia pratensis* L. (leaves) BO1, *Taraxacum officinale* Weber (young leaves) BO2, *Crepis vesicaria* subsp. *Taraxacifolia* (Thuill) Thell (young leaves) BO3, *Sonchus* spp. (young leaves) BO4, *Urtica dioica* L. (leaves) BO5, and *Cichorium intybus* L. (young leaves) BO6; and six from the Middle Agri Valley (Basilicata region, Italy), namely *Beta vulgaris* L. (leaves) MA1, *Foeniculum vulgare* Mill. (leaves, seeds and stem) MA2, *Cichorium intybus* L. (leaves) MA3, *Sonchus* spp. (leaves) MA4, *Sambucus nigra* L. (flowers) MA5, and *Asparagus acutifolius* L. (shoots) MA6. The species were selected based on the reported use of their cooking water as medicinal preparation or their higher relative frequency of citation (RFC) index, which expresses the number of informants who cited a specific wild food plant divided by the total number of informants [6, 7]. In the case of *Sonchus* spp. (BO4 and MA4), it was not possible to identify the exact species, as the informants (non-expert people who still retain traditional local knowledge on wild food plants uses) were not able to distinguish among similar plant species, which are mostly collected at the rosette stage when still lacking flowers. Therefore, more than one species was identified as a single ethnospecies and collected and analyzed together [6, 7]. Later, it was proven that the BO4 sample included both *Sonchus asper* L. and *S*. *arvensis* L. [6], while the MA4 sample included *Sonchus oleraceus* L., *S*. *asper* L. and *S*. *arvensis* L. [7]. Species voucher specimens of all used plants had been deposited at the Herbarium of the University of Bologna during previous studies [6, 7].

Several individual plants of the same species were collected simultaneously from different parts of the same sampling area and pooled together. This procedure minimized individual characteristics and allowed us to obtain homogeneous samples for all following analyses. Samples were frozen in liquid nitrogen and were stored at -80°C.

### Sample preparation

**Fig 1:**
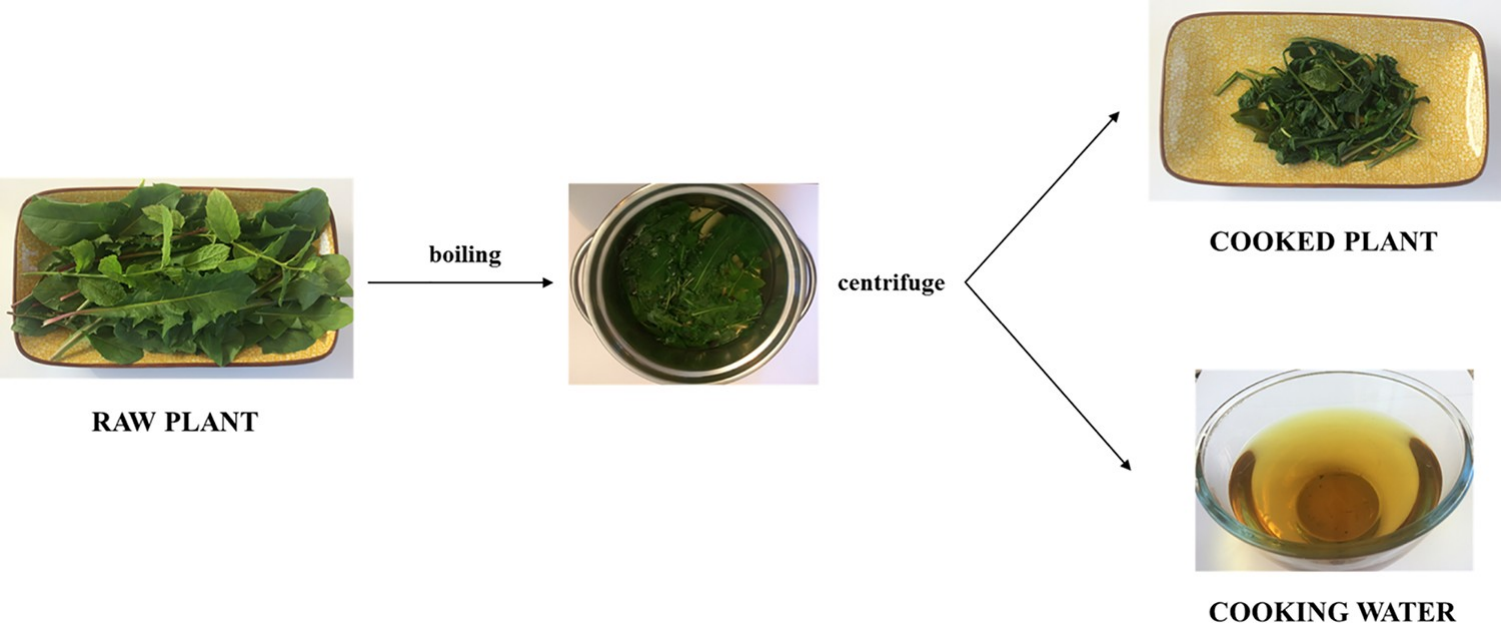
Plant sample processing. Photographs by A. Tassoni.

Plant samples were ground under liquid nitrogen with ceramic mortar and pestle. The powders were divided into aliquots which were then differently processed before analysis (Fig 1). To obtain data on raw plants, aliquots of frozen sample powder were analyzed according to the different analysis methods. In parallel, 0.3 g fresh weight (g FW) powder was incubated at 100°C in 3 mL MilliQ water for 10 minutes and then centrifuged at 4,500 *g* for 10 minutes at room temperature: liquid fractions represented cooking water samples, while solid fractions represented cooked plants.

### Spectrophotometric analysis

Raw and cooked plant samples (0.3 g FW) were extracted by overnight shaking at room temperature in 3 mL of 95% (v/v) methanol and, subsequently, centrifuged at 4,500 *g* for 10 minutes at room temperature. Obtained methanolic (raw and cooked plant samples) and aqueous extracts (cooking water) were used to assess protein, polyphenol and flavonoid contents and antioxidant activity. Protein content of extracts was determined as described by Lowry [26]. The results were expressed as mg of bovine serum albumin equivalent (BSA) per g of fresh weight (mg BSA eq /g FW) by means of a dose-response calibration curve (between 0 and 200 μg of BSA). Total polyphenol content of extracts was determined using the Folin-Ciocalteu method [27]. The results were expressed as mg of gallic acid equivalents per g of fresh weight (mg GA eq /g FW) by means of a dose-response calibration curve (between 0 and 15 μg of gallic acid). Total flavonoid content of the extracts was determined as described by Ferri et al. [27]. The results were expressed as mg of catechin equivalents per g of fresh weight (mg CAT eq /g FW) by means of a dose-response calibration curve (between 2 and 14 μg of catechin). Antioxidant activity of samples (raw and cooked plant methanolic extracts and cooking water) was measured using the ABTS method [27]. ABTS working solution (1 mL) was added to different aliquots of the samples (or the standard), and after incubation at 30°C in the dark for 30 minutes, absorbance was measured at 734 nm. The results were expressed as grams of ascorbic acid (AA) equivalents per g of fresh weight (g AA eq /g FW) by means of a dose-response calibration curve (between 0 and 2 μg of AA).

### Quantification of polyphenols by HPLC-DAD

Polyphenols were extracted from 1 mL of raw and cooked plant extracts and from cooking water. Aqueous extracts were processed without any further manipulation, while in the case of raw and cooked plant samples, methanol was evaporated and replaced with water. The samples were loaded onto a Strata-X column (33 μm polymeric sorbent, 60 mg / 3 mL, Phenomenex, Torrence, CA, USA) and polyphenols were eluted with 100% (v/v) methanol, completely dried and resuspended in 200 μL of 1:9 acetonitrile/0.2% (v/v) acetic acid before being injected into the HPLC-DAD (column Gemini C18, 5 μm particles 250 x 4.6 mm, pre-column SecurityGuard Ea, Phenomenex, Torrence CA, USA) equipped with an on-line diode array detector (MD-2010, Plus, Jasco Instruments, Großumstad, Germany) [28]. The adopted HPLC-DAD separation procedure allowed for the simultaneous analysis of 31 compounds by means of a 45 min dynamic gradient in which eluents were acetonitrile and 0.2% acetic acid [29]. The HPLC standards were purchased from Sigma-Aldrich (Milano, Italy) except for *cis*-resveratrol (cRESV), *trans*- and *cis*-resveratroloside (tRDE, cRDE), and *trans*- and *cis*-piceid (tPIC, cPIC) which were obtained as reported by Ferri et al. [28]. In particular, tRDE and tPIC were extracted from *Polygonum cuspidatum* roots, while cRESV, cRDE and cPIC were obtained by isomerization after UV irradiation. Standard compounds were injected at several concentrations (between 2 and 50 μM) to verify retention time and spectrum for peak identification and to assess the linearity range for quantification. For each plant samples, five chromatograms obtained at different wavelengths (270, 285, 305, 323 and 365 nm) were analyzed to determine the concentration of single compounds, depending on their maximum absorbance. In detail: gallic (GA), protocatechuic (PROTA), syringic (SIRA), vanillic (VANA) and *trans*-cinnamic (CINA) acids, epigallocatechin (EGC), catechin (CAT), epicatechin (EC), epicatechin gallate (ECG), epigallocatechin-gallate (EGCG), and vanillin (VAN) were quantified at 270 nm; cRESV, cPIC, cRDE and naringenin (NAR) at 285 nm; *trans*-resveratrol (tRESV), tPIC, tRDE and *p*-coumaric acid (COUMA) at 305 nm; chlorogenic (CHLORA), caffeic (CAFA), *trans*-ferulic (FERA), sinapic (SINA) acids, apigenin (API) and piceatannol (PICEAT) at 323 nm; rutin (RUT), myricetin (MYR), quercetin (QUERC), kaempferol (KAE), luteolin (LUT) and luteolin-7-glucoside (LUT-7-glu) at 365 nm (chemical structures of detected polyphenols and representative chromatograms are shown in S1 Fig).

### Quantification of biogenic amines

Free, perchloric acid (PCA) soluble-bound and PCA insoluble-bound biogenic amines (TRYPT, tryptamine; DAP, diamine-propane; CAD, cadaverine; PUT, putrescine; HIS, histamine; TYR, tyramine; SPD, spermidine; and SPM, spermine) were quantified in raw and cooked plants and in cooking water [30] (chemical structures of detected biogenic amines and representative chromatograms are shown in S2 Fig). Raw and cooked plant samples (0.2 g FW of powders) were homogenized in 1.8 mL of 4% (v/v) cold PCA and centrifuged at 15,200 *g* for 30 min at 4°C. The supernatant was separated from the pellet which was washed three times and resuspended to the original volume with 4% PCA. Triplicates of this suspension and of the supernatant of raw and cooked plants were hydrolyzed with 6 N HCl in flame-sealed glass ampoules at 110°C for 20 h in order to release polyamines from their conjugates. Aliquots (0.2 mL) of supernatant and cooking water (free polyamines), hydrolyzed supernatant (PCA-soluble conjugated polyamines) and hydrolyzed pellet (PCA-insoluble-conjugated polyamines) were derivatized with dansyl-chloride (5 mg/mL of acetone, Sigma-Aldrich, Milan, Italy), extracted with toluene and analyzed by high-performance liquid chromatography (HPLC, Jasco, Großumstad, Germany; equipped with an on-line spectrofluorometer Jasco 821-FP) with a reverse phase C18 column (Gemini, 5 μm particle diameter, 4.6 × 250 mm, Phenomenex, Torrance, CA, USA) [30]. The solvent gradient (1 mL/min) was as reported in Tassoni et al. [31]. Standard compounds (Sigma-Aldrich, Milan, Italy) were injected at several concentrations (between 160 and 1600 pmol) to verify retention time for peak identification and to assess linearity range for quantification.

### Statistical analysis

All spectrophotometric assay procedures and HPLC-DAD analyses were performed in duplicate in two technical replicates each. Biogenic amines HPLC-fluorometer analyses were performed in triplicate in three technical replicates. The results are expressed as the mean (n = 2 or n = 3) ± SD per g FW (raw and cooked samples) or per g/mL (cooking water samples). Data were tested for normality using the Shapiro-Wilk normality test, and for homogeneity using the Levene’s test for homogeneity of variance with default parameters from the package *car* (https://CRAN.R-project.org/package=car). Since data resulted parametric, the differences in protein, polyphenol, and flavonoid content and antioxidant activity, determined by spectrophotometric analysis, and in total polyphenol and biogenic amines content, determined by HPLC, were evaluated among the 12 analyzed species using a one-way ANOVA test (S1 Table) followed by a Tukey HDS test. One-way ANOVA statistical differences among individual polyphenols and among individual biogenic amines determined by HPLC are reported in S2 and S3 Tables. All statistical analyses were performed using R software version 3.5.1 (R Core Team, Vienna, Austria). Spearman’s correlation coefficients and related *p* values (S4 Table) were calculated with the program Excel.

## Results and discussion

### Sample general characterization

An overview of sample composition was initially obtained via spectrophotometric techniques as total levels of different metabolite families were quantified by specific assays.

**Fig 2:**
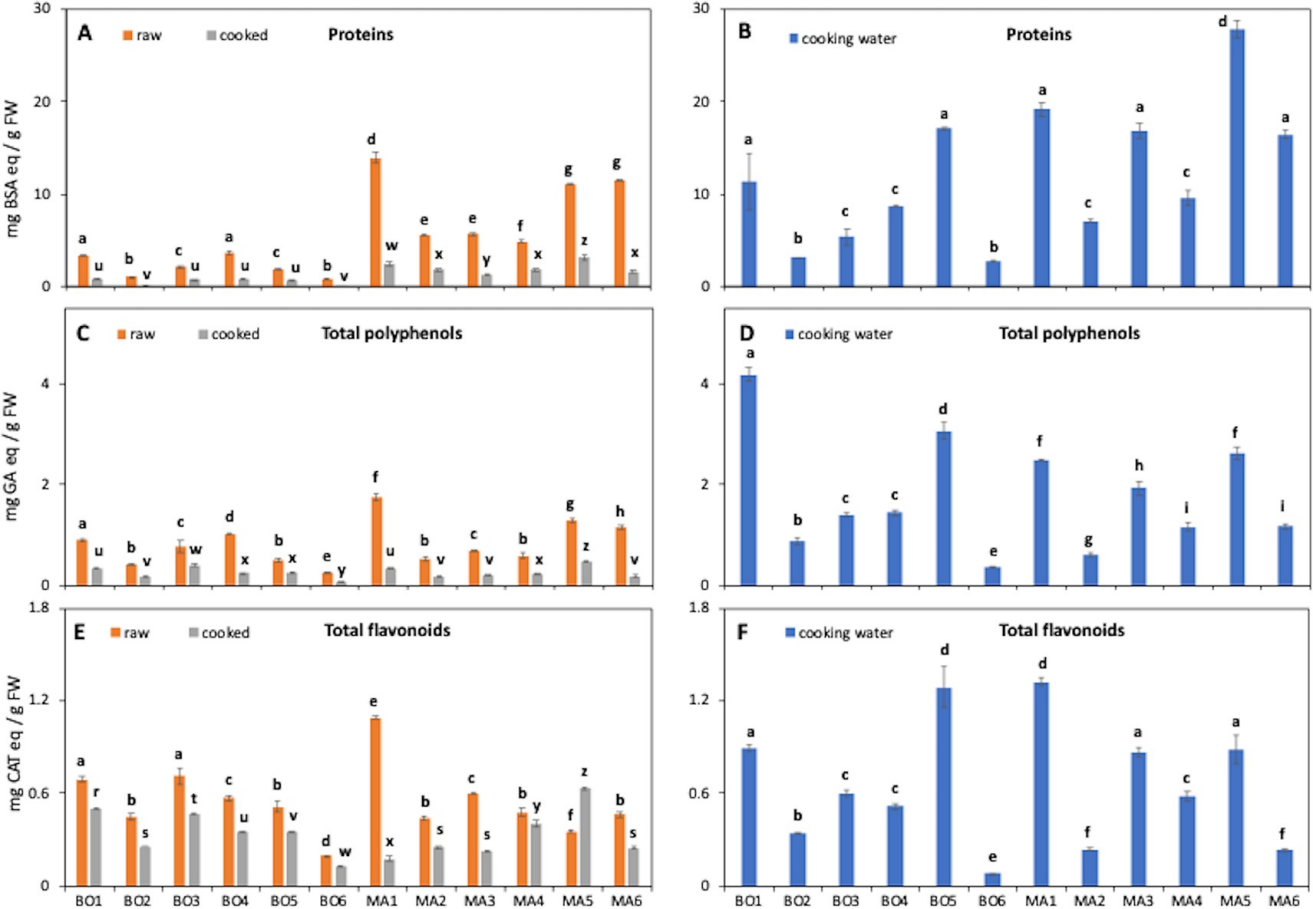
Total amounts of proteins (A, B), polyphenols (C, D) and flavonoids (E, F). Species from Bologna: *S*. *pratensis* L. (BO1), *T*. *officinalis* Weber (BO2), *C*. *vesicaria* subsp. *taraxacifolia* (BO3), *Sonchus* spp. (BO4), *U*. *dioica* L. (BO5), *C*. *intybus* L. (BO6); species from the Middle Agri Valley: *B*. *vulgaris* L. (MA1), *F*.*-vulgare* Mill. (MA2), *C*. *intybus* L. (MA3), *Sonchus* spp. (MA4), *S*. *nigra* L. (MA5), *A*. *acutifolius* L. (MA6). BSA, bovine serum albumin; GA, gallic acid; CAT, catechin. Different letters indicate a statistically significant difference (one-way ANOVA, *p* < 0.05, followed by a post hoc Tukey HSD test) among the same type of data of all species within the same treatment. Detailed one-way ANOVA statistical analysis are reported in S1 Table. Data are the mean ± SD (n = 2).

Protein content of raw and cooked samples (methanol extracts) and of cooking water are shown in Fig 2A and 2B. On average, raw samples had a 4.6-fold higher protein content than cooked samples. Overall, the highest protein concentrations were detected in cooking water of *S*. *nigra* L. (MA5, 27.8 mg BSA eq/g FW), *B*. *vulgaris* L. (MA1, 19.2 mg BSA eq /g FW) and *U*. *dioica* L. (BO5, 17.1 mg BSA eq /g FW).

As expected, polyphenol concentration was generally higher in raw samples than in cooked samples (on average 3.4-fold) (Fig 2C), in agreement with findings on some edible leaves [32], on different types of green beans [33] and on beetroot [34]. Polyphenols were mostly released into the cooking water (Fig 2D) with highest contents observed in *S*. *pratensis* L. (BO1, 4.2 mg GA eq /g FW), *U*. *dioica* L. (BO5, 3.1 mg eq GA eq /g FW) and *S*. *nigra* L. (MA5, 2.6 mg GA eq /g FW), for which a high polyphenol content had previously been demonstrated [1, 35–37]. The highest flavonoid levels were found in raw samples of *B*. *vulgaris* L. (MA1, 1.1 mg CAT eq/g FW), *S*. *pratensis* L. and *C*. *vesicaria* subsp. *taraxacifolia* (BO1 and BO3, about 0.7 mg CAT eq /g FW), in cooked plants of BO1, BO3 and MA5 (about 0.6 mg CAT eq /g FW) (Fig 2E), and in cooking water of BO5 and MA1 (about 1.3 mg CAT eq /g FW) (Fig 2F). A recent study [32] pointed out that flavonoid content in several edible leaves was increased by boiling, possibly due to an enhanced availability for extraction and to a more efficient release from intracellular proteins and altered cell wall structures. In addition, a higher solubility of phenolics with increasing temperature has been reported for *S*. *nigra* L. [38]. Similar effects could also have occurred in the present study, explaining the high flavonoid content of cooking water (Fig 2F). Data on total polyphenol and flavonoid contents of *Sonchus* spp. and *C*. *intybus* L. used in Calabrian (Southern Italy) folk tradition have previously been reported by Marrelli et al. [39]. In agreement with the present results (Fig 2C and 2E), these authors measured higher metabolite levels in *Sonchus* with respect to *Cichorium*, with significant differences due to the collection site.

### Quantification of polyphenols by HPLC

**Fig 3:**
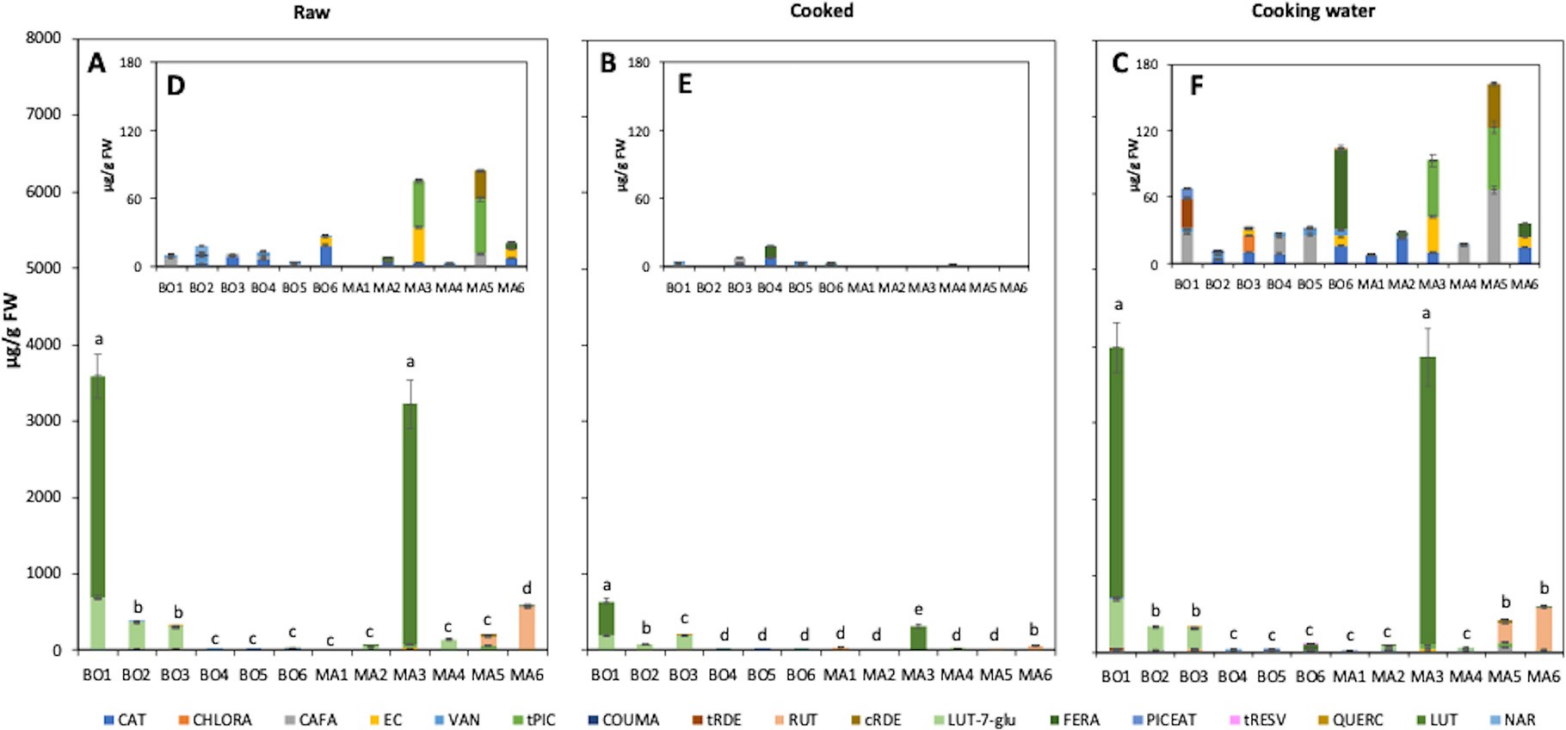
Content of individual polyphenols in raw (A, D) and cooked (B, E) methanolic plant extracts, and in cooking water (C, F). A-C, total levels; D-F: inserts showing compounds detected at lower concentration. Species from Bologna: *S*. *pratensis* L. (BO1), *T*. *officinalis* Weber (BO2), *C*. *vesicaria* subsp. *taraxacifolia* (BO3), *Sonchus* spp. (BO4), *U*. *dioica* L. (BO5), *C*. *intybus* L. (BO6); species from the Middle Agri Valley: *B*. *vulgaris* L. (MA1), *F*. *vulgare* Mill. (MA2), *C*. *intybus* L. (MA3), *Sonchus* spp. (MA4), *S*. *nigra* L. (MA5), *A*. *acutifolius* L. (MA6). Caffeic acid, CAFA; chlorogenic acid, CHLORA; *p*-coumaric acid, COUMA; *trans*-ferulic acid, FERA; catechin, CAT; *cis*-resveratroloside, cRDE; epicatechin, EC; luteolin, LUT; luteolin-7-glucoside, LUT-7-glu; naringenin, NAR; piceatannol, PICEAT; rutin, RUT; *trans*-piceid, tPIC; *trans*-resveratroloside, tRDE; *trans*-resveratrol, tRESV; quercetin, QUERC; vanillin, VAN. Different lower-case letters in indicate a statistically significant difference (one-way ANOVA, *p* < 0.05, followed by post hoc Tukey HSD test) among total polyphenol levels of all species within the same treatment. Detailed one-way ANOVA statistical analysis of total and individual compound levels are reported in S1 and S2 Tables. Data are the mean ± SD (n = 2).

The detailed polyphenolic profile of raw and cooked plant samples and of cooking water was determined by HPLC-DAD (Fig 3). Seventeen different compounds were detected among hydroxycinnamic acids (caffeic acid, CAFA; chlorogenic acid, CHLORA; *p*-coumaric acid, COUMA; *trans*-ferulic acid, FERA), phenolic aldehydes (vanillin, VAN), stilbenes (*cis*-resveratroloside, cRDE; piceatannol, PICEAT; *trans*-piceid, tPIC; *trans*-resveratroloside, tRDE; *trans*-resveratrol) and flavonoid subclasses, such as flavones (luteolin, LUT; luteolin-7-glucoside, LUT-7-glu), flavonols (quercetin, QUERC; rutin, RUT), flavanones (naringenin, NAR), and flavanols (catechin, CAT; epicatechin, EC) (S1 Fig). LUT, LUT-7-glu and RUT were present at 100 to 1000-times higher levels (Fig 3A–3C) than the average of other identified compounds (Fig 3D–3F). As expected, lower levels of polyphenols were generally detected in cooked samples with respect to raw plants. Considering the compounds present at lower concentration, the content of raw samples was on average 2.4-fold higher than that of cooked samples in Bologna plants, while, on average, cooking water samples showed 2.6-fold and 17.5-fold higher levels than raw and cooked samples, respectively (Fig 3D–3F). Overall, higher levels of the lower-level polyphenols were detected in BO6 and MA5 cooking water extracts (Fig 3F).

The most abundant phenolic compounds were the flavones LUT and LUT-7-glu in *S*. *pratensis* L. (BO1) raw and cooking water samples (respectively, 637.2 and 3278.6 μg /g FW), and LUT in *C*. *intybus* L. (MA3) raw plants and cooking water (respectively, 3148.6 and 3768.8 μg /g FW) (Fig 3A, and 3C). The flavonol RUT was only detected in raw, cooked and cooking water samples of *S*. *nigra* L. (MA5) and *A*. *acutifolius* L. (MA6) (Fig 3A–3C). In general, most of the identified polyphenols were released into the cooking water (Fig 3C and 3F), consistent with total phenol spectrophotometric quantification (Fig 2D). Previously published reports on some of the plant species here analyzed [1] seem to be coherent with the present data on flavonoids and phenolic acids (Fig 3). Specifically, LUT and LUT-7-glu have been previously identified as major component in *Crepis* spp., LUT-7-glu and CAFA in *Sonchus* spp. [40], CAFA and coumaric acid (CUMA) in *U*. *dioica* leaves [36], and RUT and CAFA in *S*. *nigra* L. berries and flowers [37, 38].

Changes in phenolic compounds upon cooking plants were previously observed with generally lower levels being found with respect to raw material [23, 33]. On one hand, the thermal degradation of molecules reduces their concentration, whereas on the other hand matrix softening due to cell wall destruction increases the extractability of phytochemicals, resulting in a higher concentration compared to raw material [23]. Moreover, cooking can alter the bioavailability and the consequent intestinal absorption of polyphenols. For example, it was recently demonstrated that open pan boiling drastically reduced the uptake of quercetin (QUERC) from cooked onion in the Caco-2 cell *in vitro* system [41]. Dietary flavonoids show positive effects on human health by exerting antioxidant and/or other biological activities. In the analyzed plant species, LUT was the compound detected at the highest concentration, in particular in *S*. *pratensis* L. (BO1) and *C*. *intybus* L. (MA3) (Fig 3A–3C). These data are particularly interesting from a nutraceutical point of view because LUT is known for its antioxidant, anti-inflammatory and anti-cancer activities [42]. According to both total flavonoid quantification and HPLC analysis (Figs 2 and 3), LUT was mostly released from the plant to the water during the cooking process. It is also known that heat treatment may influence the structure of flavonoids, often leading to changes in their bioactivities. For example, El Gueder and colleagues [42] suggested that heat treatment lowers the anti-tumor potential of LUT.

The present research also showed different polyphenol profiles for *Sonchus* spp. (BO4, MA4) and, in particular, *C*. *intybus* L. (BO6, MA3) when collected from different study areas (Fig 3). This is in agreement with previously published findings reporting diverse metabolite patterns in samples of the same species collected from different sites, such as *C*. *intybus* L. and *S*. *asper* L. [39], 15 Spanish wild edible plants [43] and *U*. *dioica* leaves [36]. In the latter study, the differences in the phenolic compound profiles and contents were ascribed to the environmental diversity of the growing areas, such as climate, nutrient richness and light.

It has, however, to be considered that, from a chemical point of view, cooking water samples were plant aqueous extracts, while raw and cooked samples were methanolic extracts obtained either from the raw plant or from the pellet after water extraction of cooked samples. These extraction steps are common in phytochemical analyses of plant samples and it has been demonstrated that aqueous-alcoholic mixtures are the most efficient solvents and that extraction conditions and polarity of the solvents lead to different yields of specific compound families [44–46]. In the present case, the methanolic extraction was aimed at obtaining as much as possible the total range of phytochemicals possibly ingested by consumers of alimurgic plants. Consequently, the differences observed among raw, cooked and cooking water samples (Figs 2 and 3) may be partially ascribed to the polarity of the extraction solvent (methanol or water).

### Biogenic amines

**Fig 4:**
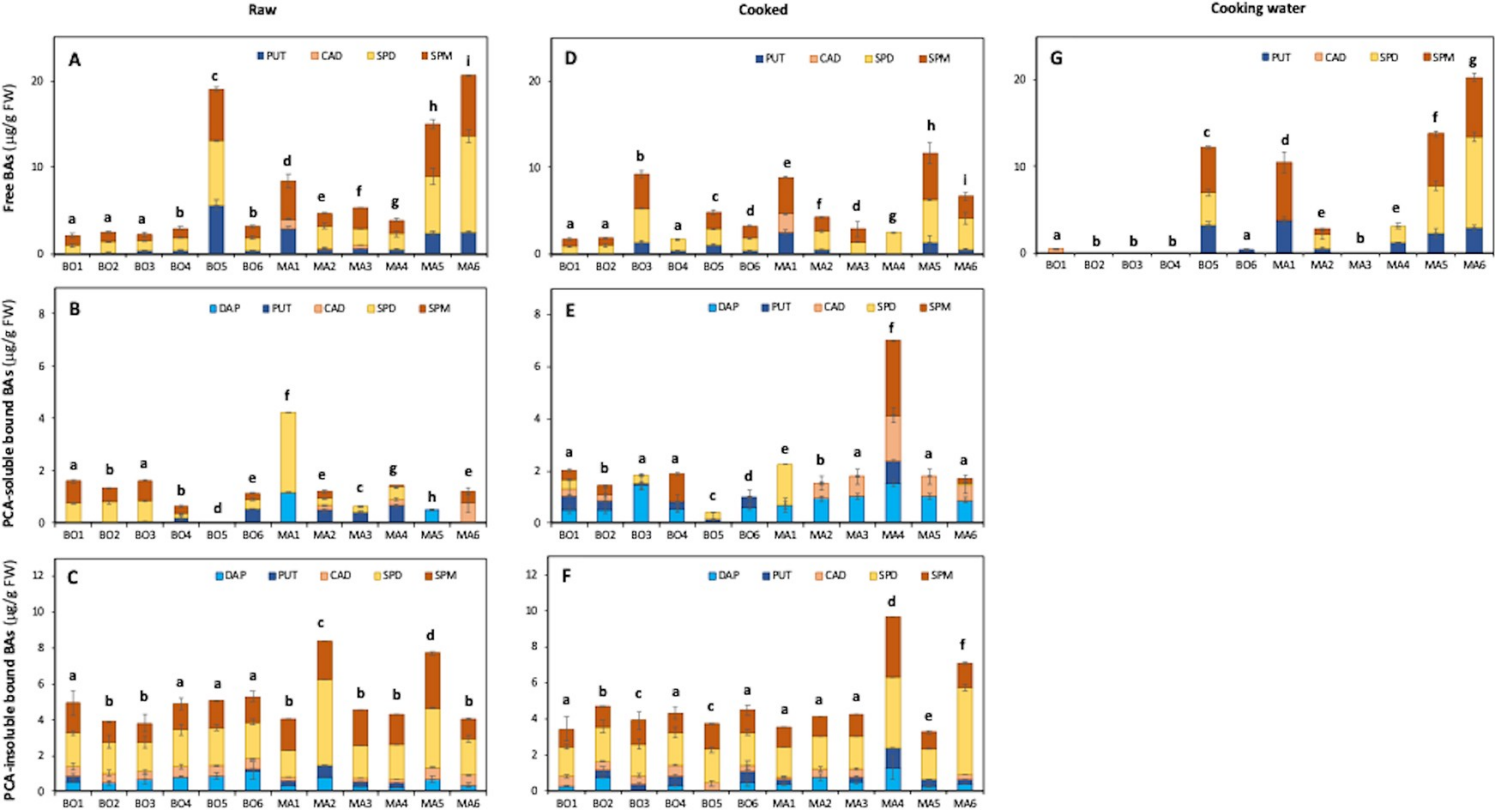
Content of free (A, D, G), PCA-soluble bound (B, E) and PCA-insoluble bound (C, F) biogenic amines in raw (A, B, C) and cooked (D, E, F) plants, and in cooking water (G) samples. Species from Bologna: *S*. *pratensis* L. (BO1), *T*. *officinalis* Weber (BO2), *C*. *vesicaria* subsp. *taraxacifolia* (BO3), *Sonchus* spp. (BO4), *U*. *dioica* L. (BO5), *C*. *intybus* L. (BO6); species from the Middle Agri Valley: *B*. *vulgaris* L. (MA1), *F*. *vulgare* Mill. (MA2), *C*. *intybus* L. (MA3), *Sonchus* spp. (MA4), *S*. *nigra* L. (MA5), *A*. *acutifolius* L. (MA6). Cadaverine, CAD; diamine-propane, DAP; putrescine, PUT; spermidine, SPD; spermine, SPM. Different letters indicate a statistically significant difference (one-way ANOVA, *p* < 0.05, followed by a post hoc Tukey HSD test) of all species within the same treatment and the same type of data (free, PCA-soluble and PCA-insoluble biogenic amines). Detailed one-way ANOVA statistical analysis of total and individual compound levels are reported in S1 and S3 Tables. Data are the mean ± SD (n = 3).

Free, perchloric acid (PCA)-soluble and PCA-insoluble mono- and polyamine levels were determined by HPLC-fluorometry of raw and cooked plants and of cooking water samples (Fig 4, S2 Fig). Up to now, very few published papers have reported a change in biogenic amine (BA) levels after cooking vegetables and, most importantly, these data only referred to their free forms [33, 47]. According to the authors’ knowledge, the present study is the first report on the effect of cooking on the amount of PCA soluble- and insoluble-bound BAs in wild alimurgic plants.

In the analyzed edible species, spermidine (SPD) and spermine (SPM) were the most abundant free BAs, ranging from an average of 75.4% of the total content in cooking water (Fig 4G) up to an average of 83.7% and 87.5%, respectively, in raw and cooked samples (Fig 4A and 4D). In general, raw samples had a higher total free BA concentration than cooking water (up to 8.6-fold in BO6) and cooked plants (up to 3.1-fold in MA6). The plants having the highest content of total free BAs were BO5 (19.0 μg /g FW) and MA6 (20.6 μg /g FW). Free form of diamine-propane (DAP) was not detected in any sample, while the monoamines tryptamine, tyramine and histidine were not detected in any form in all plants. Compared to some common food vegetables, the analyzed wild alimurgic species contained 4- to 200-fold [33, 47, 48] lower amounts of free BA in raw samples, and up to about 60-fold lower amounts in boiled plants [33, 47].

PCA soluble and insoluble-bound BA levels were only determined for raw and cooked samples (Fig 4B, 4C, 4E and 4F) as it was assumed that cooking water only contained free BAs. Cooked samples showed on average 1.6-fold higher levels of PCA soluble-bound amines than raw plants (Fig 4B and 4E) with the highest total content found in raw *B*. *vulgaris* L. (MA1, 4.3 μg /g FW) and cooked *Sonchus* spp. (MA4, 7.0 μg /g FW). In contrast to free BA data, DAP was the most abundant PCA soluble-bound polyamine in almost all cooked species (up to 77.4% of the total content in *C*. *vesicaria*, BO3). Among the PCA insoluble-bound BAs (Fig 4C and 4F), the sum of SPD and SPM represented on average the 76.3% and 75.8% of total BAs, respectively, in raw and cooked plants. No statistically significant differences in PCA insoluble-bound BA yields were generally found among species, except for raw plants of *F*. *vulgare* Mill. (MA2) and *S*. *nigra* L. (MA5) and cooked plants of *Sonchus* spp. (MA4) and *A*. *acutifolius* L. (MA6), which had, respectively, up to 2.1 and 2.5-folds higher levels than other species. Overall, the highest total BA amount was detected in *A*. *acutifolius* L. (MA6, 25.8 and 35.7 μg /g FW, respectively, in raw plant and in cooked plant + cooking water) and in *U*. *dioica* (BO5, 24.1 and 21.1 μg /g FW, respectively, in raw plant and in cooked plant + cooking water). In general, the total BA amount of cooked alimurgic plants resulted to be comparable or, in a few cases, slightly higher (BO3, MA4) than that of raw plants. Therefore, these results seem to indicate that, at least for the analyzed wild food species, the cooking process does not cause thermal degradation of BAs, as previously shown for other phytochemicals [23], and may sometimes enhance the bioavailability of their conjugated forms from the food matrix, in accordance with previous published papers on common food vegetables [33, 47].

In addition, in some samples of cooking water (*S*. *pratensis* L. (BO1), *T*. *officinalis* Weber (BO2), *C*. *vesicaria* subsp. *taraxacifolia* (BO3), *C*. *intybus* L. (MA3)), BAs were found to be absent or present at very low levels. These data seem to be very interesting as for these very same species the traditional dietary use of cooking water for therapeutic purposes (e.g., as digestive or diuretic) has been reported in previous ethnobotanical studies [6, 7].

### Antioxidant activity

The antioxidant activity of raw and cooked plants and of cooking waters was spectrophotometrically determined by means of ABTS assay (Table 1). In agreement with the data on total polyphenols and flavonoids (Fig 2D and 2F), cooking water samples showed the highest antioxidant activity (on average, 2.5 and 6.6-fold higher than, respectively, raw and cooked plants). Again, the plants with the highest activity were *S*. *pratensis* L. (BO1, 3.57 mg AA eq /g FW in cooking water), *U*. *dioica* L. (BO5, 3.65 mg AA eq /g FW) and *S*. *nigra* L. (MA5, 3.43 mg AA eq /g FW). Similarly to the polyphenol data (Figs 2 and 3), antioxidant activity of the species common in both collection areas, *Sonchus* spp. (BO4 and MA4) and *C*. *intybus* L. (BO6 and MA3), resulted slightly affected by the site of collection (Table 1).

**Table 1:** Antioxidant activity expressed as mg of ascorbic acid (AA) equivalent per g of fresh weight (mg AA eq /g FW).

**Plants (Bologna)**	**Raw**	**Cooked**	**Cooking water**
*Salvia pratensis* L. (BO1)	0.95 ± 0.01 ^a^	0.47 ± 0.02 ^a^	3.57 ± 0.49 ^a^
*Taraxacum officinalis* Weber (BO2)	0.54 ± 0.04 ^b^	0.23 ± 0.01 ^b^	1.56 ± 0.04 ^b^
*Crepis vesicaria* subsp. *taraxacifolia* (BO3)	0.73 ± 0.01 ^b^	0.43 ± 0.01 ^a^	1.88 ± 0.07 ^b^
*Sonchus* spp. (BO4)	0.82 ± 0.04 ^b^	0.26 ± 0.01 ^b^	1.46 ± 0.10 ^b^
*Urtica dioica* L. (BO5)	0.75 ± 0.19 ^b^	0.34 ± 0.05 ^a^	3.65 ± 0.07 ^a^
*Cichorium intybus* L. (BO6)	0.36 ± 0.04 ^c^	0.14 ± 0.01 ^c^	0.37 ± 0.02 ^d^
**Plants (Middle Agri Valley)**	**Raw**	**Cooked**	**Cooking water**
*Beta vulgaris* L. (MA1)	2.06 ± 0.04 ^d^	0.35 ± 0.03 ^a^	3.05 ± 0.18 ^c^
*Foeniculum vulgare* Mill. (MA2)	0.61 ± 0.02 ^b^	0.25 ± 0.01 ^b^	0.88 ± 0.20 ^e^
*Cichorium intybus* L. (MA3)	0.65 ± 0.03 ^b^	0.23 ± 0.02 ^b^	2.75 ± 0.01 ^c^
*Sonchus* spp. (MA4)	0.67 ± 0.01 ^b^	0.38 ± 0.08 ^a^	1.68 ± 0.11 ^b^
*Sambucus nigra* L. (MA5)	1.72 ± 0.07 ^e^	0.62 ± 0.01 ^d^	3.43 ± 0.14 ^a^
*Asparagus acutifolius* L. (MA6)	1.68 ± 0.03 ^e^	0.23 ± 0.01 ^b^	1.68 ± 0.14 ^b^

Different lower-case letters indicate a statistically significant difference (one-way ANOVA, *p* < 0.05, followed by post hoc Tukey HSD test) among all plant species within the same treatment. Detailed one-way ANOVA statistical analysis are reported in S1 Table. Data are the mean ± SD (n = 2).

Boiling reduced the antioxidant properties of all plants analyzed up to a maximum of 7.3-times in *A*. *acutifolius* L. (MA6, Table 1). Similar results have been found in previous studies, e.g. a 30% reduction in antioxidant activity (DPPH assay) after green bean boiling or steaming [33] and a 34% and 54% reduction (ABTS and DPPH assays, respectively) in red beetroot flesh [34]. According to the authors, this reduction may principally be ascribed to the leaching of compounds into the water during cooking. In addition, in cooked plants the original substances may have been transformed into differently active compounds.

### Correlation analysis

The potential relationships among quantified metabolites (Figs 2 and 4) and antioxidant capacity (Table 1) were investigated by correlation analysis (Table 2) and couples of variables with a strong relationship (coefficient absolute value > 0.8) were identified. In general, protein, total polyphenol, total flavonoid and antioxidant activity show a strong, positive and statistically relevant (S4 Table) association with each other in all samples. In particular, as expected, antioxidant activity was strongly correlated to polyphenol, flavonoid and protein content, proving that all these metabolite families include antioxidant compounds (correlation coefficients > 0.8, *p* < 0.05, Table 2 and S4 Table), as was previously demonstrated for *S*. *officinalis* L. leaves [35], *C*. *intybus* L. and *S*. *asper* L. aerial parts [39] and *S*. *nigra* L. flowers [37]. Conversely, total BAs were not or weakly correlated (positively or negatively) with all the other compound families (Table 2 and S4 Table).

**Table 2:** Correlation coefficients between the analyzed variables: Proteins, polyphenols, flavonoids and antioxidant activity (spectrophotometric analysis, Fig 2, Table 1), total biogenic amines (free, PCA-soluble- and PCA-insoluble bound, HPLC analysis, Fig 4). *P* values and level of significance of correlation coefficients are reported in S4 Table.

	Variables	Bologna					Middle Agri Valley				
**Cooking water**		Proteins	Polyphenols	Flavonoids	Biogenic amines	Antioxidant activity	Proteins	Polyphenols	Flavonoids	Biogenic amines	Antioxidant activity
	Proteins		0.81*	0.95*	0.81*	0.87*		0.89*	0.58	0.53	0.90*
	Polyphenols			0.84*	0.42	0.94*			0.88*	0.23	0.99*
	Flavonoids				0.77	0.95*				-0.09	0.85*
	Biogenic amines					0.61					0.20
	Antioxidant activity										
**Raw plant**	Proteins		0.96*	0.71	-0.15	0.89*		0.97*	0.50	0.74	0.99*
	Polyphenols			0.82*	-0.27	0.88*			0.63	0.58	0.97*
	Flavonoids				-0.10	0.88*				-0.18	0.45
	Biogenic amines					0.09					0.75
	Antioxidant activity										
**Cooked plant**	Proteins		0.82*	0.87*	0.18	0.78		0.95*	0.66	0.47	0.91*
	Polyphenols			0.97*	0.52	0.96*			0.74	0.43	0.95*
	Flavonoids				0.31	0.97*				0.55	0.89*
	Biogenic amines					0.36					0.59
	Antioxidant activity										

* strongly correlated (absolute coefficient value > 0.8).

## Conclusions

The present study aimed at investigating the phytochemical content and antioxidant capacity of 12 traditionally consumed wild plants from two different Italian areas (Bologna, Northern Italy, and Middle Agri Valley, Southern Italy). As most of these species were in the past eaten both raw or after boiling and the cooking water was sometimes used as food-medicine, the purpose of the present research was to analyze the biochemical profiles of raw, cooked and cooking water plant samples according to past traditional uses.

The data on total polyphenols and flavonoids showed that a large amount of these compounds was released into the cooking water which showed, accordingly, also the highest levels of antioxidant activity. Detailed polyphenolic profiling led to the identification of 17 different compounds of which LUT, LUT-7-glu and RUT were 100 to 1000-times more abundant than the average level of other phenols. Free and conjugated (PCA soluble- and insoluble-bound) biogenic amine profiles were also determined making the present study, according to the authors’ knowledge, the first report on the effect of cooking on biogenic amine contents in alimurgic food plants. Most interestingly, biogenic amines were absent or present at very low levels in cooking water of those species (e.g., *S*. *pratensis* L., *T*. *officinalis* Weber, *C*. *vesicaria* subsp. *taraxacifolia* and *C*. *intybus* L.) of which traditionally a decoction is used for therapeutic purposes.

In conclusion, the presented data indicate that, depending on their phytochemical profiles and antioxidant levels, the analyzed wild food plant species could exert different effects on consumers’ health, thus confirming their use (and in particular that of their cooking water) as food-derived medicinal tool for improving human health, in accordance with past local popular traditions.

## Supporting information

S1 Fig Representative polyphenol HPLC-DAD chromatograms indicating the peak assignment of each metabolite. (A-N) standards and (O-Z) samples. Chemical structures of the identified compounds (formulas were drown using ChemSketch, ACD Labs) (AA-AD). Five chromatograms obtained at different wavelengths (270, 285, 305, 323 and 365 nm) were analyzed for each sample to determine the concentration of single compounds, depending on their maximum absorbance. (O) *Cichorium intybus* L. (BO6) cooking water chromatogram at 270 nm. (P) *Sambucus nigra* L. (MA5) cooking water at 285 nm. (Q) *Taraxacum officinalis* Weber (BO2) raw at 285 nm. (R) *Sambucus nigra* L. (MA5) cooking water at 305 nm. (S) *Sambucus nigra* L. (MA5) cooking water at 305 nm. (T) *Salvia pratensis* L. (BO1) cooking water at 305 nm. (U) *Cichorium intybus* L. (BO6) cooking water chromatogram at 305 nm. (V) *Crepis vesicaria* subsp. *taraxacifolia* (BO3) cooking water at 323 nm. (W) *Salvia pratensis* L. (BO1) cooking water at 323 nm. (X) *Cichorium intybus* L. (BO6) cooking water chromatogram at 323 nm. (Y) *Crepis vesicaria* subsp. *taraxacifolia* (BO3) cooked at 365 nm. (Z) *Salvia pratensis* L. (BO1) cooking water at 365 nm. (AA) Identified flavonoids chemical structures. (AB) Identified hydroxycinnamic acids chemical structures. (AC) Identified phenolic aldehydes chemical structures. (AD) Identified stilbenes chemical structures.
(PDF)

S2 Fig (A) HPLC-fluorometer chromatograms of detected biogenic amine (BAs) standard mixture and (B) of PCA-soluble bound BAs in cooked sample of *Salvia pratensis* L. (BO1) (sample example). (C) Chemical structures of detected BAs. Formulas were drawn using ChemSketch (ACD Labs).
(PDF)

S1 Table One-way ANOVA statistical analysis output related to data presented in Fig 2 (total levels of proteins, polyphenols and flavonoids determined by spectrophotometric analysis), Fig 3 (total levels of polyphenols determined by HPLC-DAD), Fig 4 (total levels of free, PCA soluble-bound and PCA insoluble-bound biogenic amines determined by HPLC-fluorometer), Table 1 (antioxidant activity determined by spectrophotometric analysis). DF, degrees of freedom, SS, sum of squares, MS, mean of squares. Significance codes: (***), *p* < 0.001; (**), *p* < 0.01; (*), *p* < 0.05.
(PDF)

S2 Table One-way ANOVA statistical analysis output data related to individual polyphenol levels determined by HPLC-DAD and shown in Fig 3. CAFA, caffeic acid; CHLORA, chlorogenic acid; COUMA, p-coumaric acid; FERA, trans-ferulic acid; CAT, catechin; cRDE, cis-resveratroloside; EC, epicatechin; LUT, luteolin; LUT-7-glu, luteolin-7-glucoside; NAR, naringenin; PICEAT, piceatannol; RUT, rutin; tPIC, trans-piceid; tRDE, trans-resveratroloside; tRESV, trans-resveratrol; QUERC, quercetin; VAN, vanillin. DF, degrees of freedom, SS, sum of squares, MS, mean of squares. Significance codes: (***), *p* < 0.001; (**), *p* < 0.01; (*), *p* < 0.05.
(PDF)

S3 Table One-way ANOVA statistical analysis output related to individual biogenic amine levels determined by HPLC-fluorometer and shown in Fig 4. DAP, diamine-propane; CAD, cadaverine; PUT, putrescine; SPD, spermidine; SPM, spermine. DF, degrees of freedom, SS, sum of squares, MS, mean of squares. Significance codes: (***), *p* < 0.001; (**), *p* < 0.01; (*), *p* < 0.05.
(PDF)

S4 Table *P* values and level of significance of correlation coefficients reported in Table 2. The values were calculated by means of Excel programme. Significance codes: (***), *p* < 0.001; (**), *p* < 0.01; (*), *p* < 0.05.
(PDF)
