# Supplementary material for: Phytochemical characterization of raw and cooked traditionally consumed alimurgic plants
Source: PLoS One. 2021 Aug 26;16(8):e0256703. doi: 10.1371/journal.pone.0256703 (PMC8389401; doi:10.1371/journal.pone.0256703)
Supplement: S1 Fig — (A-N) standards and (O-Z) samples. Chemical structures of the identified compounds (formulas were drown using ChemSketch, ACD Labs) (AA-AD). Five chromatograms obtained at different wavelengths (270, 285, 305, 323 and 365 nm) were analyzed for each sample to determine the concentration of single compounds, depending on their maximum absorbance. (O) Cichorium intybus L. (BO6) cooking water chromatogram at 270 nm. (P) Sambucus nigra L. (MA5) cooking water at 285 nm. (Q) Taraxacum officinalis Weber (BO2) raw at 285 nm. (R) Sambucus nigra L. (MA5) cooking water at 305 nm. (S) Sambucus nigra L. (MA5) cooking water at 305 nm. (T) Salvia pratensis L. (BO1) cooking water at 305 nm. (U) Cichorium intybus L. (BO6) cooking water chromatogram at 305 nm. (V) Crepis vesicaria subsp. taraxacifolia (BO3) cooking water at 323 nm. (W) Salvia pratensis L. (BO1) cooking water at 323 nm. (X) Cichorium intybus L. (BO6) cooking water chromatogram at 323 nm. (Y) Crepis vesicaria subsp. taraxacifolia (BO3) cooked at 365 nm. (Z) Salvia pratensis L. (BO1) cooking water at 365 nm. (AA) Identified flavonoids chemical structures. (AB) Identified hydroxycinnamic acids chemical structures. (AC) Identified phenolic aldehydes chemical structures. (AD) Identified stilbenes chemical structures. (PDF) [file pone.0256703.s001.pdf]

Figure S1. Representative polyphenol HPLC-DAD chromatograms indicating the peak assignment of each metabolite: (A-N) standards and (O-Z) samples. Chemical structures of the identified compounds (formulas were drawn using ChemSketch, ACD Labs) (AA-AD). Five chromatograms obtained at different wavelengths (270, 285, 305, 323 and 365 nm) were analyzed for each sample to determine the concentration of single compounds, depending on their maximum absorbance. (O) *Cichorium intybus* L. (BO6) cooking water chromatogram at 270 nm. (P) *Sambucus nigra* L. (MA5) cooking water at 285 nm. (Q) *Taraxacum officinalis* Weber (BO2) raw at 285 nm. (R) *Sambucus nigra* L. (MA5) cooking water at 305 nm. (S) *Sambucus nigra* L. (MA5) cooking water at 305 nm. (T) *Salvia pratensis* L. (BO1) cooking water at 305 nm. (U) *Cichorium intybus* L. (BO6) cooking water chromatogram at 305 nm. (V) *Crepis vesicaria* subsp. *taraxacifolia* (BO3) cooking water at 323 nm. (W) *Salvia pratensis* L. (BO1) cooking water at 323 nm. (X) *Cichorium intybus* L. (BO6) cooking water chromatogram at 323 nm. (Y) *Crepis vesicaria* subsp. *taraxacifolia* (BO3) cooked at 365 nm. (Z) *Salvia pratensis* L. (BO1) cooking water at 365 nm. (AA) Identified flavonoids chemical structures. (AB) Identified hydroxycinnamic acids chemical structures. (AC) Identified phenolic aldehydes chemical structures. (AD) Identified stilbenes chemical structures.

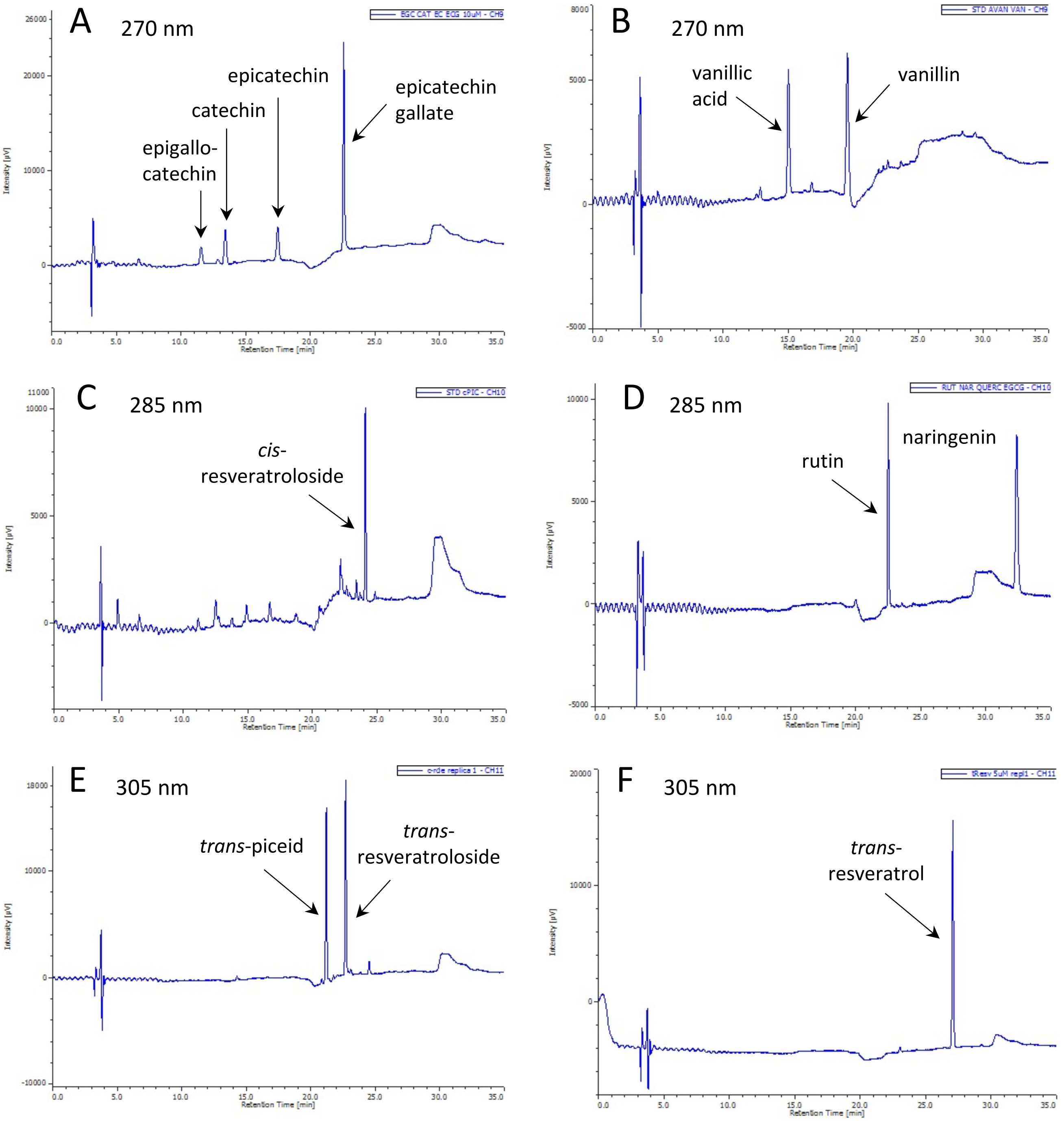

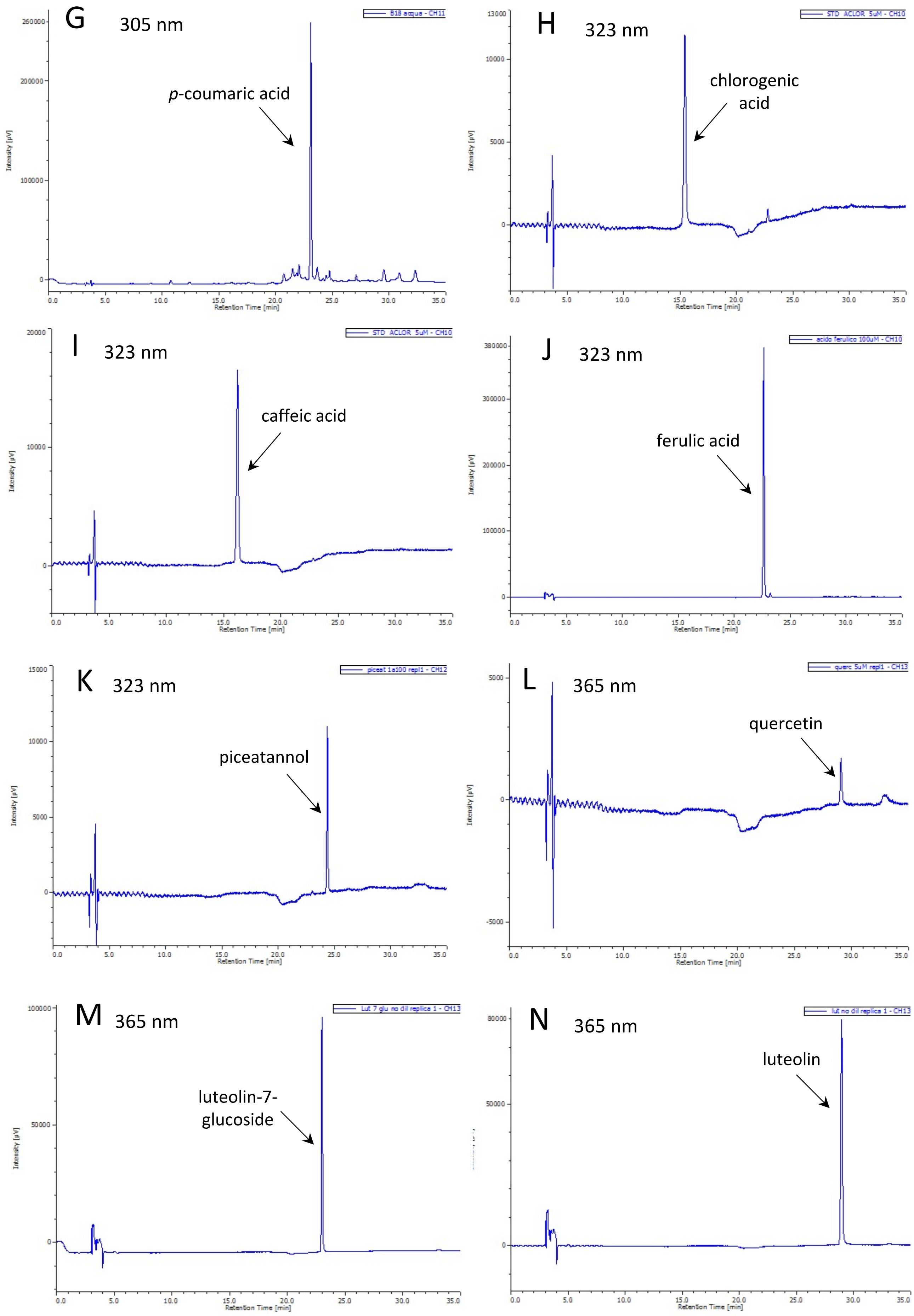
G
305 nm
B18 acqua - CH11
p-coumaric acid
H
323 nm
STD_ACLOR_5uM - CH10
chlorogenic acid
I
323 nm
STD_ACLOR_5uM - CH10
caffeic acid
J
323 nm
acido ferulico 100uM - CH10
ferulic acid
K
323 nm
piceat 1a100 repl1 - CH12
piceatannol
L
365 nm
querc 5uM repl1 - CH13
quercetin
M
365 nm
Lut 7 glu no dil replica 1 - CH13
luteolin-7-glucoside
N
365 nm
lut no dil replica 1 - CH13
luteolin
Intensity [µV]
Retention Time [min]

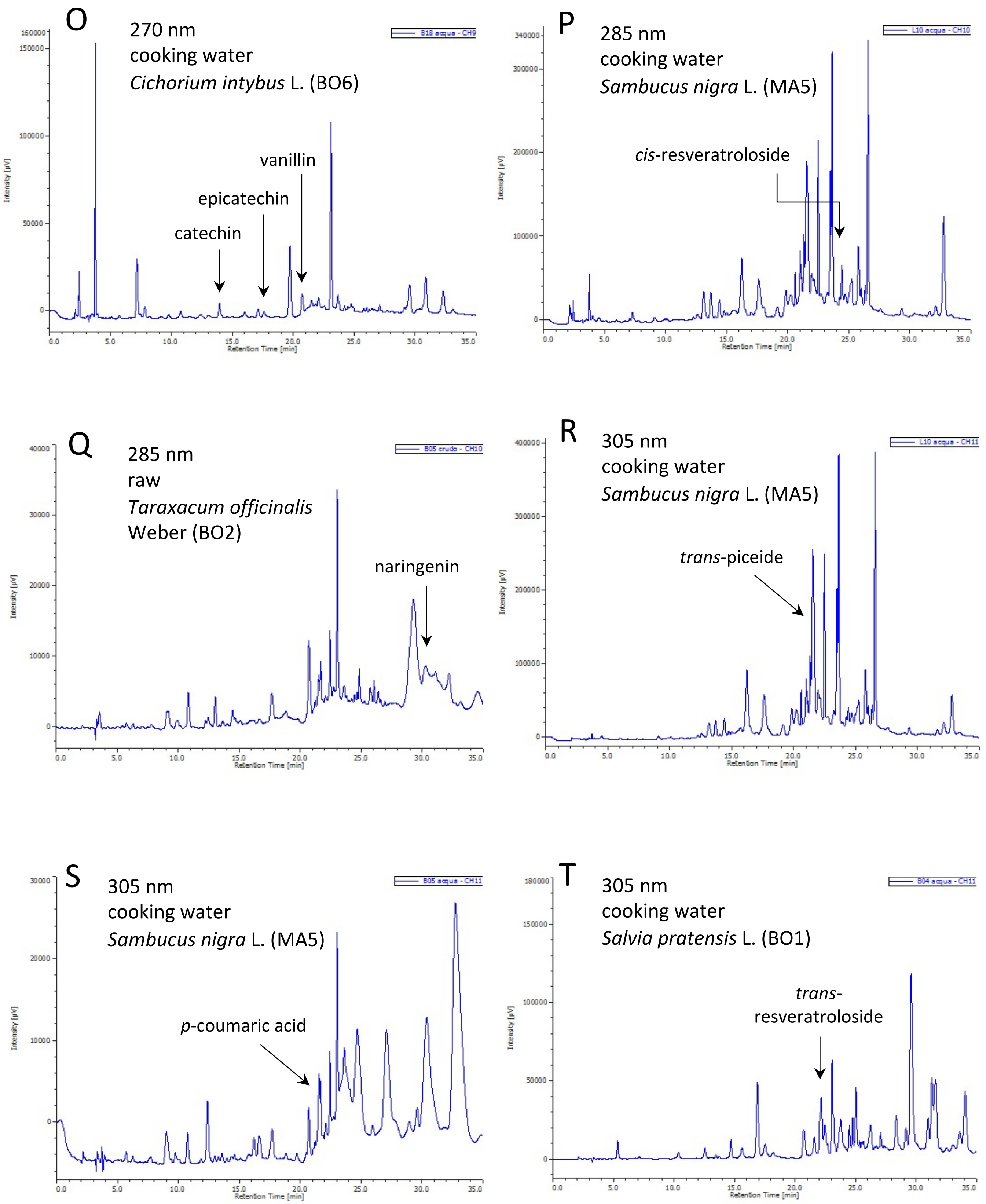
O
270 nm
cooking water
Cichorium intybus L. (BO6)
B18 acqua - CH9
catechin
epicatechin
vanillin
Intensity [µV]
Retention Time [min]
P
285 nm
cooking water
Sambucus nigra L. (MA5)
L10 acqua - CH10
cis-resveratroloside
Intensity [µV]
Retention Time [min]
Q
285 nm
raw
Taraxacum officinalis
Weber (BO2)
B05 crudo - CH10
naringenin
Intensity [µV]
Retention Time [min]
R
305 nm
cooking water
Sambucus nigra L. (MA5)
L10 acqua - CH11
trans-piceide
Intensity [µV]
Retention Time [min]
S
305 nm
cooking water
Sambucus nigra L. (MA5)
B05 acqua - CH11
p-coumaric acid
Intensity [µV]
Retention Time [min]
T
305 nm
cooking water
Salvia pratensis L. (BO1)
B04 acqua - CH11
trans-
resveratroloside
Intensity [µV]
Retention Time [min]

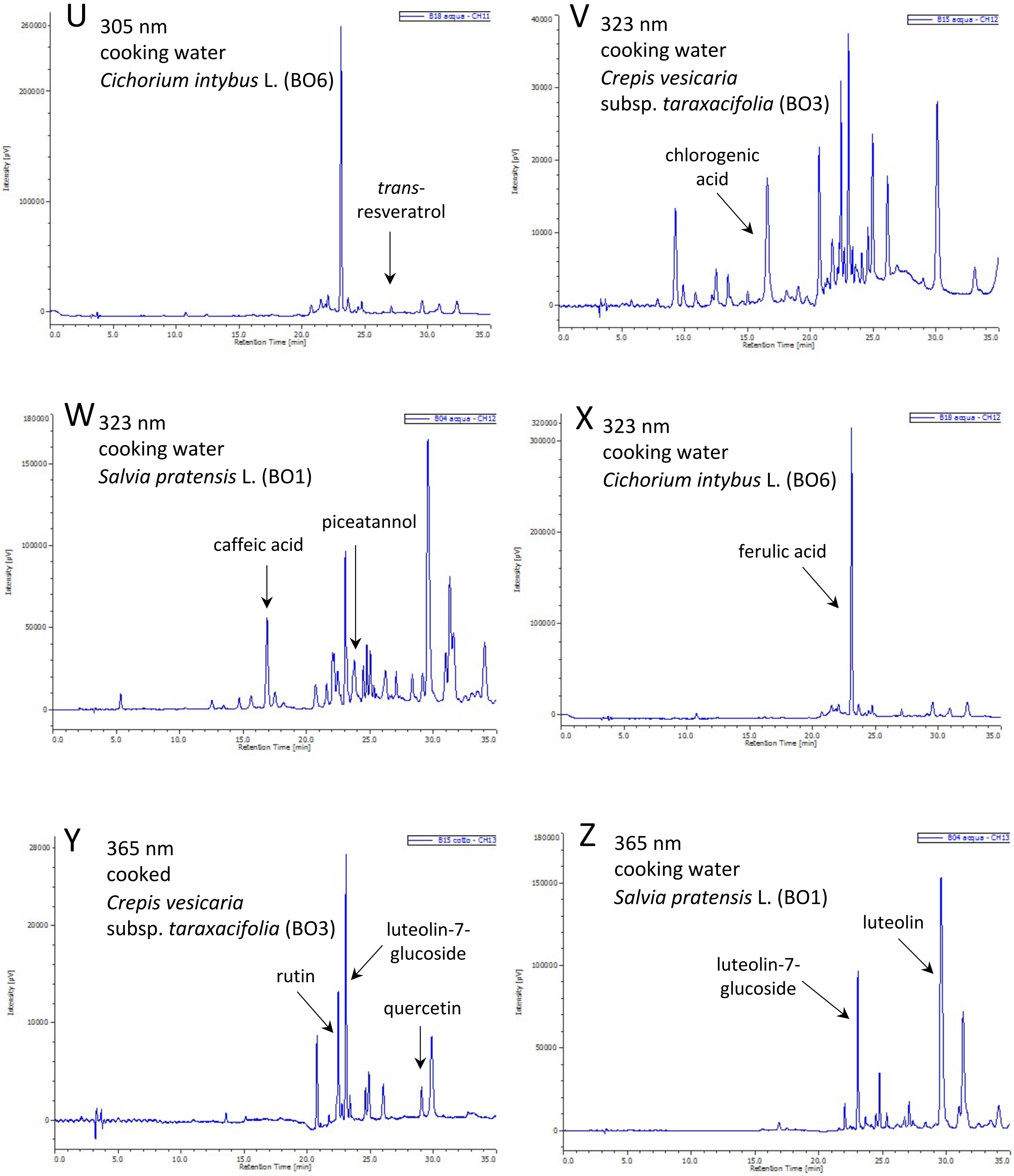
U
305 nm
cooking water
Cichorium intybus L. (BO6)
trans-
resveratrol
B18 acqua - CH11
Intensity [µV]
Retention Time [min]
V
323 nm
cooking water
Crepis vesicaria
subsp. *taraxacifolia* (BO3)
chlorogenic
acid
B15 acqua - CH12
Intensity [µV]
Retention Time [min]
W
323 nm
cooking water
Salvia pratensis L. (BO1)
caffeic acid
piceatannol
B04 acqua - CH12
Intensity [µV]
Retention Time [min]
X
323 nm
cooking water
Cichorium intybus L. (BO6)
ferulic acid
B18 acqua - CH12
Intensity [µV]
Retention Time [min]
Y
365 nm
cooked
Crepis vesicaria
subsp. *taraxacifolia* (BO3)
rutin
luteolin-7-
glucoside
quercetin
B15 cotto - CH13
Intensity [µV]
Retention Time [min]
Z
365 nm
cooking water
Salvia pratensis L. (BO1)
luteolin-7-
glucoside
luteolin
B04 acqua - CH13
Intensity [µV]
Retention Time [min]

AA

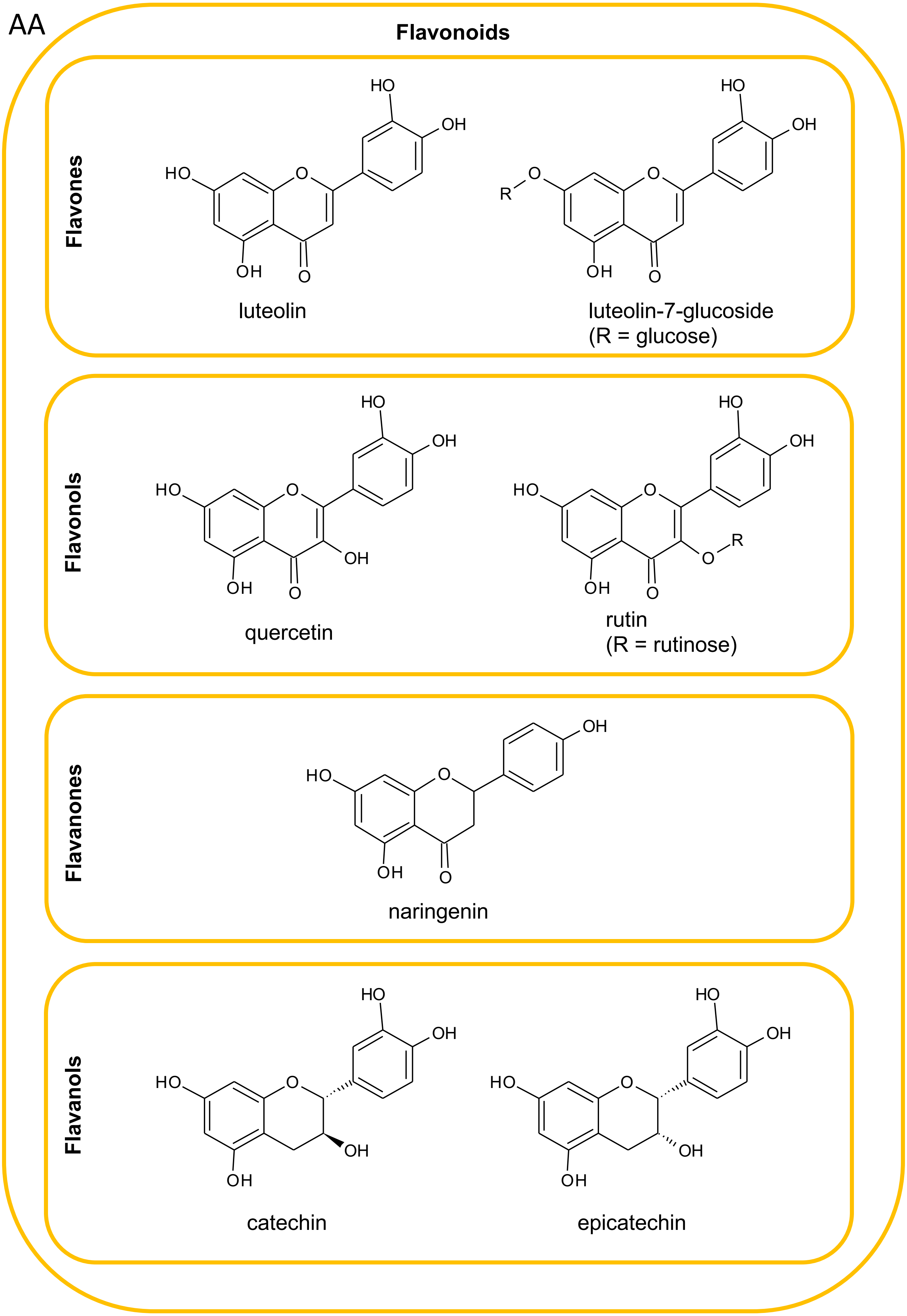
Flavonoids
Flavones
luteolin
luteolin-7-glucoside
(R = glucose)
Flavonols
quercetin
rutin
(R = rutinose)
Flavanones
naringenin
Flavanols
catechin
epicatechin

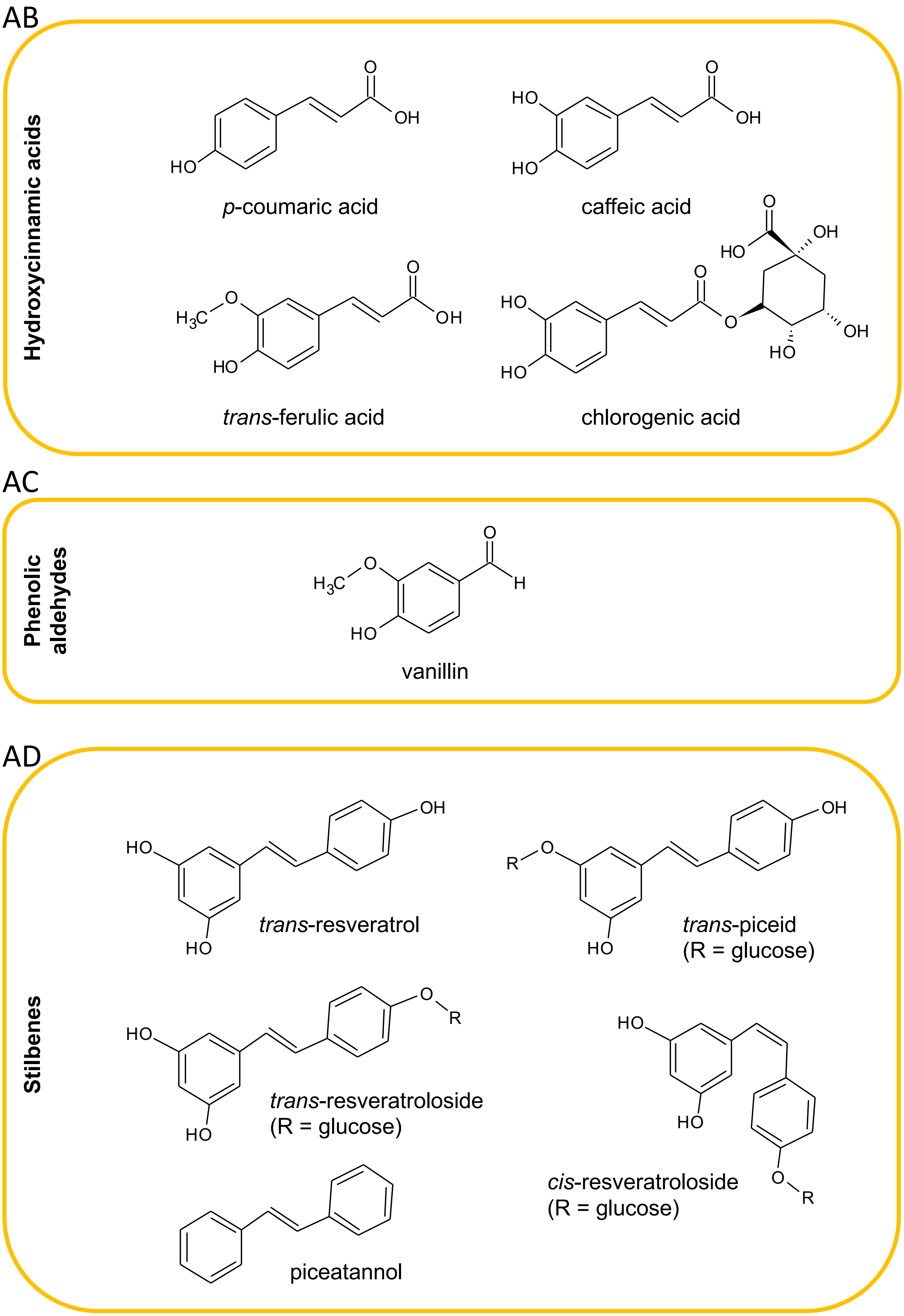
AB
Hydroxycinnamic acids
p-coumaric acid
caffeic acid
trans-ferulic acid
chlorogenic acid
AC
Phenolic aldehydes
vanillin
AD
Stilbenes
trans-resveratrol
trans-piceid
(R = glucose)
trans-resveratroloside
(R = glucose)
cis-resveratroloside
(R = glucose)
piceatannol
