# Supplementary material for: Phytochemical characterization of raw and cooked traditionally consumed alimurgic plants
Source: PLoS One. 2021 Aug 26;16(8):e0256703. doi: 10.1371/journal.pone.0256703 (PMC8389401; doi:10.1371/journal.pone.0256703)

Figure S2. (A) HPLC-fluorometer chromatograms of detected biogenic amine (BAs) standard mixture and (B) of PCA-soluble bound BAs in cooked sample of *Salvia pratensis* L. (BO1) (sample example). (C) Chemical structures of detected BAs. Formulas were drawn using ChemSketch (ACD Labs).

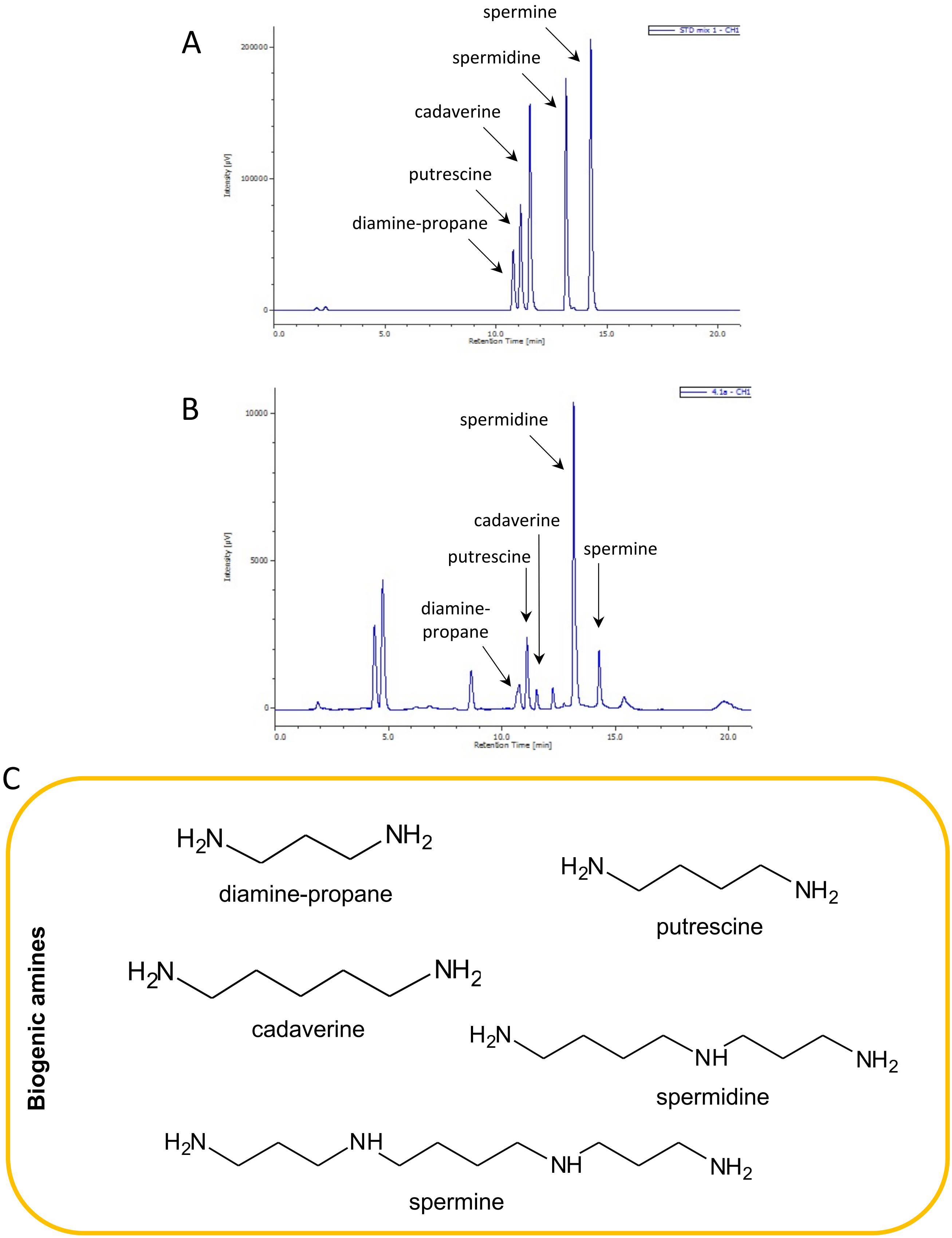

Supplement: S2 Fig — (A) HPLC-fluorometer chromatograms of detected biogenic amine (BAs) standard mixture and (B) of PCA-soluble bound BAs in cooked sample of Salvia pratensis L. (BO1) (sample example). (C) Chemical structures of detected BAs. Formulas were drawn using ChemSketch (ACD Labs). (PDF) [file pone.0256703.s002.pdf]
