# Supplementary material for: Phytochemical characterization of raw and cooked traditionally consumed alimurgic plants
Source: PLoS One. 2021 Aug 26;16(8):e0256703. doi: 10.1371/journal.pone.0256703 (PMC8389401; doi:10.1371/journal.pone.0256703)
Supplement: S1 Table — DF, degrees of freedom, SS, sum of squares, MS, mean of squares. Significance codes: (***), p < 0.001; (**), p < 0.01; (*), p < 0.05. (PDF) [file pone.0256703.s003.pdf]

S1 Table. One-way ANOVA statistical analysis output related to data presented in Figure 2 (total levels of proteins, polyphenols and flavonoids determined by spectrophotometric analysis), Figure 3 (total levels of polyphenols determined by HPLC-DAD), Figure 4 (total levels of free, PCA soluble-bound and PCA insoluble-bound biogenic amines determined by HPLC-fluorometer), Table 1 (antioxidant activity determined by spectrophotometric analysis). DF, degrees of freedom, SS, sum of squares, MS, mean of squares. Significance codes: (***), $p < 0.001$; (**), $p < 0.01$; (*), $p < 0.05$.

**PROTEINS - Total levels (spectrophotometric analysis)**						
**Samples**		**DF**	**SS**	**MS**	**F value**	***P* value**
Raw plant (Fig. 2A)	Sample	13	891.1	68.55	1162	<2e-16 ***
	Residuals	34	2.0	0.06		
Cooked plant (Fig. 2A)	Sample	13	39.43	3.0334	270.2	<2e-16 ***
	Residuals	34	0.38	0.0112		
Cooking water (Fig. 2B)	Sample	13	2516.2	193.55	319.6	<2e-16 ***
	Residuals	34	20.6	0.61		
**POLYPHENOLS – Total levels (spectrophotometric analysis)**						
**Samples**		**DF**	**SS**	**MS**	**F value**	***P* value**
Raw plant (Fig. 2C)	Sample	13	7.963	0.6126	426.3	<2e-16 ***
	Residuals	34	0.049	0.0014		
Cooked plant (Fig. 2C)	Sample	13	0.5663	0.04356	454.2	<2e-16 ***
	Residuals	34	0.0033	0.00010		
Cooking water (Fig. 2D)	Sample	13	55.80	4.292	801.9	<2e-16 ***
	Residuals	34	0.18	0.005		
**TOTAL FLAVONOIDS - Total levels (spectrophotometric analysis)**						
**Samples**		**DF**	**SS**	**MS**	**F value**	***P* value**
Raw plant (Fig. 2E)	Sample	13	2.1749	0.16730	443	<2e-16 ***
	Residuals	34	0.0128	0.00038		
Cooked plant (Fig. 2E)	Sample	13	0.9543	0.07341	1393	<2e-16 ***
	Residuals	34	0.0018	0.00005		
Cooking water (Fig. 2F)	Sample	13	7.237	0.5567	317.9	<2e-16 ***
	Residuals	34	0.060	0.0018		
**POLYPHENOLS - Total levels (HPLC-DAD analysis)**						
**Samples**		**DF**	**SS**	**MS**	**F value**	***P* value**
Raw plant (Fig. 3A+B)	Sample	12	72335166	6027931	4756	<2e-16 ***
	Residuals	35	44356	1267		
Cooked plant (Fig. 3C+D)	Sample	12	1532152	127679	2386	<2e-16 ***
	Residuals	35	1873	54		
Cooking water (Fig. 3E+F)	Sample	12	94399907	7866659	1627	<2e-16 ***
	Residuals	35	169267	4836		
**FREE BIOGENIC AMINES – Total levels (HPLC-fluorometer analysis)**						

Samples		DF	SS	MS	F value	*P* value
Raw plant (Fig. 4A)	Sample	12	2075.6	172.96	17910	<2e-16 ***
	Residuals	35	0.3	0.01		
Cooked plant (Fig. 4D)	Sample	13	0.7776	0.05981	98.4	<2e-16 ***
	Residuals	34	0.0207	0.00061		
Cooking water (Fig.4G)	Sample	13	2220.6	170.81	24391	<2e-16 ***
	Residuals	34	0.2	0.01		
**PCA-SOLUBLE BOUND BIOGENIC AMINES – Total levels (HPLC-fluorometer analysis)**						
**Samples**		**DF**	**SS**	**MS**	**F value**	***P* value**
Raw plant (Fig. 4B)	Sample	12	50.12	4.176	8235	<2e-16 ***
	Residuals	35	0.02	0.001		
Cooked plant (Fig. 4E)	Sample	12	123.68	10.306	7072	<2e-16 ***
	Residuals	35	0.05	0.001		
**PCA INSOLUBLE-BOUND BIOGENIC AMINES – Total levels (HPLC-fluorometer analysis)**						
**Samples**		**DF**	**SS**	**MS**	**F value**	***P* value**
Raw plant (Fig. 4C)	Sample	12	100.72	8.394	2092	<2e-16 ***
	Residuals	35	0.14	0.004		
Cooked plant (Fig. 4F)	Sample	12	142.87	11.906	3138	<2e-16 ***
	Residuals	35	0.13	0.004		
**ANTIOXIDANT ACTIVITY – (spectrophotometric analysis)**						
**Samples**		**DF**	**SS**	**MS**	**F value**	***P* value**
Raw plant (Table 1)	Sample	13	13.059	1.0045	361.7	<2e-16 ***
	Residuals	34	0.094	0.0028		
Cooked plant (Table 1)	Sample	13	0.7776	0.05981	98.4	<2e-16 ***
	Residuals	34	0.0207	0.00061		
Cooking water (Table 1)	Sample	13	53.15	4.088	234.2	<2e-16 ***
	Residuals	34	0.59	0.017		
