# Supplementary material for: Phytochemical characterization of raw and cooked traditionally consumed alimurgic plants
Source: PLoS One. 2021 Aug 26;16(8):e0256703. doi: 10.1371/journal.pone.0256703 (PMC8389401; doi:10.1371/journal.pone.0256703)
Supplement: S2 Table — CAFA, caffeic acid; CHLORA, chlorogenic acid; COUMA, p-coumaric acid; FERA, trans-ferulic acid; CAT, catechin; cRDE, cis-resveratroloside; EC, epicatechin; LUT, luteolin; LUT-7-glu, luteolin-7-glucoside; NAR, naringenin; PICEAT, piceatannol; RUT, rutin; tPIC, trans-piceid; tRDE, trans-resveratroloside; tRESV, trans-resveratrol; QUERC, quercetin; VAN, vanillin. DF, degrees of freedom, SS, sum of squares, MS, mean of squares. Significance codes: (***), p < 0.001; (**), p < 0.01; (*), p < 0.05. (PDF) [file pone.0256703.s004.pdf]

S2 Table. One-way ANOVA statistical analysis output data related to individual polyphenol levels determined by HPLC-DAD and shown in Figure 3. CAFA, caffeic acid; CHLORA, chlorogenic acid; COUMA, p-coumaric acid; FERA, trans-ferulic acid; CAT, catechin; cRDE, cis-resveratroloside; EC, epicatechin; LUT, luteolin; LUT-7-glu, luteolin-7-glucoside; NAR, naringenin; PICEAT, piceatannol; RUT, rutin; tPIC, trans-piceid; tRDE, trans-resveratroloside; tRESV, trans-resveratrol; QUERC, quercetin; VAN, vanillin. DF, degrees of freedom, SS, sum of squares, MS, mean of squares. Significance codes: (***), $p < 0.001$; (**), $p < 0.01$; (*), $p < 0.05$.

**RAW PLANT SAMPLES**						
**Compound**		**DF**	**SS**	**MS**	**F value**	***P* value**
CAT	Sample	8	173.8	21.72	0.328	0.897
	Residuals	2	132.6	66.30		
CAFA	Sample	8	137.79	17.224	14.9	0.0644 #
	Residuals	2	2.31	1.156		
VAN	Sample	8	35.84	4.480	2.284	0.34
	Residuals	2	3.92	1.961		
EC	Sample	8	597.3	74.66	0.532	0.786
	Residuals	2	280.6	140.30		
tPIC	Sample	8	2461.9	307.7	0.707	0.702
	Residuals	2	870.7	435.3		
cRDE	Sample	8	570.9	71.36	2.633e+31	<2e-16 ***
	Residuals	2	0.0	0.00		
NAR	Sample	8	47.65	5.957	1.885e+32	<2e-16 ***
	Residuals	2	0.00	0.000		
COUMA	Sample	8	8.019	1.002	1.041e+32	<2e-16 ***
	Residuals	2	0.000	0.000		
FERA	Sample	8	35.4	4.425	4.487e+30	<2e-16 ***
	Residuals	2	0.0	0.000		
QUERC	Sample	8	3.111	0.3889	9.347e+66	<2e-16 ***
	Residuals	2	0.000	0.0000		
LUT	Sample	8	10057613	1257202	0.507	0.799
	Residuals	2	4956967	2478483		
LUT-7-glu	Sample	8	471024	58878	12.74	0.0748 #
	Residuals	2	9241	4621		
RUT	Sample	8	283735	35467	1.098e+31	<2e-16 ***
	Residuals	2	0	0		
**COOKED PLANT SAMPLES**						
**Compound**		**DF**	**SS**	**MS**	**F value**	***P* value**
CAT	Sample	8	24.40	3.05	0.20	0.957
	Residuals	2	29.26	14.63		
CAFA	Sample	8	4.979	0.6224	3.89	0.221

	Residuals	2	0.320	0.1600		
VAN	Sample	8	5.788	0.7236	1.253	0.517
	Residuals	2	1.155	0.5776		
COUMA	Sample	8	0.06627	0.008284	2.844e+30	<2e-16 ***
	Residuals	2	0.00000	0.000000		
FERA	Sample	8	36.70	4.587	0.187	0.966
	Residuals	2	48.93	24.464		
QUERC	Sample	8	20.77	2.596	4.249e+63	<2e-16 ***
	Residuals	2	0.00	0.000		
LUT	Sample	8	195074	24384	1.018	0.584
	Residuals	2	47883	23941		
LUT-7-glu	Sample	8	55352	6919	29.89	0.0328 *
	Residuals	2	463	231		
RUT	Sample	8	3688	461	1.169e+31	<2e-16 ***
	Residuals	2	0	0		
**COOKING WATER SAMPLES**						
**Compound**		**DF**	**SS**	**MS**	**F value**	***P* value**
CAT	Sample	8	553.5	69.19	2.376	0.33
	Residuals	2	58.2	29.12		
CAFA	Sample	8	4320	540.1	1278	0.000782 ***
	Residuals	2	1	0.4		
VAN	Sample	8	30.56	3.82	0.366	0.875
	Residuals	2	20.88	10.44		
tRESV	Sample	8	0.2887	0.03608	0.205	0.959
	Residuals	2	0.3528	0.17640		
CHLORA	Sample	8	177.7	22.21	1.55e+64	<2e-16 ***
	Residuals	2	0.0	0.00		
tRDE	Sample	8	679.5	84.94	4.641e+31	<2e-16 ***
	Residuals	2	0.0	0.00		
PICEAT	Sample	8	81.53	10.19	1.454e+32	<2e-16 ***
	Residuals	2	0.00	0.00		
EC	Sample	8	686.0	85.75	0.627	0.739
	Residuals	2	273.5	136.77		
tPIC	Sample	8	3408	425.9	0.651	0.727
	Residuals	2	1308	653.8		
cRDE	Sample	8	1429	178.7	3.84e+30	<2e-16 ***
	Residuals	2	0	0.0		
COUMA	Sample	8	11.38	1.423	5.773e+31	<2e-16 ***
	Residuals	2	0.00	0.000		
FERA	Sample	8	2102	262.7	0.197	0.962

	Residuals	2	2662	1330.8		
QUERC	Sample	8	3.011	0.3764	1.165e+63	<2e-16 ***
	Residuals	2	0.000	0.0000		
LUT	Sample	8	13335754	1666969	0.469	0.819
	Residuals	2	7101851	3550926		
LUT-7-glu	Sample	8	418346	52293	115	0.00865 **
	Residuals	2	909	455		
RUT	Sample	8	314683	39335	7.492e+30	<2e-16 ***
	Residuals	2	0	0		
