# Supplementary material for: Phytochemical characterization of raw and cooked traditionally consumed alimurgic plants
Source: PLoS One. 2021 Aug 26;16(8):e0256703. doi: 10.1371/journal.pone.0256703 (PMC8389401; doi:10.1371/journal.pone.0256703)
Supplement: S3 Table — DAP, diamine-propane; CAD, cadaverine; PUT, putrescine; SPD, spermidine; SPM, spermine. DF, degrees of freedom, SS, sum of squares, MS, mean of squares. Significance codes: (***), p < 0.001; (**), p < 0.01; (*), p < 0.05. (PDF) [file pone.0256703.s005.pdf]

S3 Table. **One-way ANOVA statistical analysis output related to individual biogenic amine levels determined by HPLC-fluorometer and shown in Figure 4.** DAP, diamine-propane; CAD, cadaverine; PUT, putrescine; SPD, spermidine; SPM, spermine. DF, degrees of freedom, SS, sum of squares, MS, mean of squares. Significance codes: (***), $p < 0.001$; (**), $p < 0.01$; (*), $p < 0.05$.

**RAW PLANT SAMPLES – Free biogenic amines**						
**Compound**		**DF**	**SS**	**MS**	**F value**	**P value**
PUT	Sample	8	56.23	7.029	19.93	0.0486 *
	Residuals	2	0.71	0.353		
CAD	Sample	8	112.52	14.065	300	0.00333 **
	Residuals	2	0.09	0.047		
SPD	Sample	8	0.5149	0.06436	4.385	0.199
	Residuals	2	0.0294	0.01468		
SPM	Sample	8	0.5777	0.07222	1.217	0.526
	Residuals	2	0.1187	0.05934		
**RAW PLANT SAMPLES – PCA-soluble bound biogenic amines**						
DAP	Sample	8	0.2259	0.02824	2.189e+31	<2e-16 ***
	Residuals	2	0.0000	0.00000		
PUT	Sample	8	28.971	3.621	199.8	0.00499 **
	Residuals	2	0.036	0.018		
CAD	Sample	8	0.1101	0.01377	0.206	0.959
	Residuals	2	0.1339	0.06696		
SPD	Sample	8	112.52	14.065	300	0.00333 **
	Residuals	2	0.09	0.047		
SPM	Sample	8	56.23	7.029	19.93	0.0486 *
	Residuals	2	0.71	0.353		
**RAW PLANT SAMPLES – PCA-insoluble bound biogenic amines**						
DAP	Sample	8	0.2259	0.02824	2.189e+31	<2e-16 ***
	Residuals	2	0.0000	0.00000		
PUT	Sample	8	0.5777	0.07222	1.217	0.526
	Residuals	2	0.1187	0.05934		
CAD	Sample	8	0.5149	0.06436	4.385	0.199
	Residuals	2	0.0294	0.01468		
SPD	Sample	8	0.956	0.11950	4.344	0.201
	Residuals	2	0.055	0.02751		
SPM	Sample	8	0.8948	0.1119	3.485	0.242
	Residuals	2	0.0642	0.0321		
**COOKED PLANT SAMPLES – Free biogenic amines**						
**Compound**		**DF**	**SS**	**MS**	**F value**	**P value**
PUT	Sample	8	4.1	0.5125	1.529e+33	<2e-16 ***

	Residuals	2	0.0	0.0000		
CAD	Sample	8	19.808	2.4760	20.92	0.0464 *
	Residuals	2	0.237	0.1183		
SPD	Sample	8	30.566	3.821	6.773	0.135
	Residuals	2	1.128	0.564		
SPM	Sample	8	1.8085	0.22606	2.571	0.31
	Residuals	2	0.1759	0.08793		
**COOKED PLANT SAMPLES – PCA soluble-bound biogenic amines**						
DAP	Sample	8	0.6908	0.08635	0.721	0.696
	Residuals	2	0.2395	0.11974		
PUT	Sample	8	2.5536	0.3192	1.366	0.489
	Residuals	2	0.4674	0.2337		
CAD	Sample	8	35.20	4.400	6.344	0.143
	Residuals	2	1.39	0.694		
SPD	Sample	8	7.229	0.9036	3.059	0.27
	Residuals	2	0.591	0.2954		
SPM	Sample	8	1.2677	0.15847	3.853	0.222
	Residuals	2	0.0823	0.04113		
**COOKED PLANT SAMPLES – PCA insoluble-bound biogenic amines**						
DAP	Sample	8	0.8070	0.1009	0.832	0.65
	Residuals	2	0.2426	0.1213		
PUT	Sample	8	0.4032	0.05040	4.53	0.193
	Residuals	2	0.0222	0.01112		
CAD	Sample	8	1031.0	128.88	122.8	0.0081 **
	Residuals	2	2.1	1.05		
SPD	Sample	8	5.499	0.6874	89.83	0.0111 *
	Residuals	2	0.015	0.0077		
SPM	Sample	8	7.229	0.9036	3.059	0.222
	Residuals	2	0.0823	0.04113		
**COOKING WATER SAMPLES – Free biogenic amines**						
**Compound**		**DF**	**SS**	**MS**	**F value**	**P value**
PUT	Sample	8	0.3477	0.04347	2.277	0.341
	Residuals	2	0.0382	0.01909		
CAD	Sample	8	0.4429	0.05537	0.961	0.604
	Residuals	2	0.1153	0.05763		
SPD	Sample	8	11.397	1.4247	68.29	0.0145 *
	Residuals	2	0.042	0.0209		
SPM	Sample	8	2.9779	0.3722	3.091	0.267
	Residuals	2	0.2409	0.1204		
