# Supplementary material for: Phytochemical characterization of raw and cooked traditionally consumed alimurgic plants
Source: PLoS One. 2021 Aug 26;16(8):e0256703. doi: 10.1371/journal.pone.0256703 (PMC8389401; doi:10.1371/journal.pone.0256703)
Supplement: S4 Table — The values were calculated by means of Excel programme. Significance codes: (***), p < 0.001; (**), p < 0.01; (*), p < 0.05. (PDF) [file pone.0256703.s006.pdf]

Table S4. *P* values and level of significance of correlation coefficients reported in Table 2. The values were calculated by means of Excel programme. Significance codes: (***), $p < 0.001$; (**), $p < 0.01$; (*), $p < 0.05$.

	Variables	Bologna					Middle Agri Valley				
		Proteins	Polyphenols	Flavonoids	Biogenic amines	Antioxidant activity	Proteins	Polyphenols	Flavonoids	Biogenic amines	Antioxidant activity
**Cooking water**	Proteins		0.0324*	0.0008***	0.0124*	0.0129*		0.0077**	0.2061	0.1980	0.0059**
	Polyphenols			0.0212*	0.2221	0.0013**			0.0092**	0.5874	0.00001***
	Flavonoids				0.0206*	0.0010**				0.9044	0.0177*
	Biogenic amines					0.0866					0.6196
	Antioxidant activity										
**Raw plant**	Proteins		0.0004***	0.0904	0.9192	0.0075**		0.0002***	0.2935	0.1920	0.00001***
	Polyphenols			0.0283*	0.8492	0.0108*			0.1561	0.2581	0.0002***
	Flavonoids				0.8301	0.0095**				0.6228	0.3546
	Biogenic amines					0.4603					0.1480
	Antioxidant activity										
**Cooked plant**	Proteins		0.0274*	0.0134*	0.7093	0.0464*		0.0007***	0.1277	0.8269	0.0046**
	Polyphenols			0.0002***	0.1283	0.0006***			0.0073**	0.8122	0.0012**
	Flavonoids				0.2508	0.0002***				0.6326	0.0082**
	Biogenic amines					0.1803					0.8041
	Antioxidant activity										
